# Crystal structures of di­chlorido­palladium(II), -platinum(II) and -rhodium(III) complexes containing 8-(di­phenyl­phosphan­yl)quinoline

**DOI:** 10.1107/S2056989015006076

**Published:** 2015-04-02

**Authors:** Takayoshi Suzuki, Hiroshi Yamaguchi, Masayuki Fujiki, Akira Hashimoto, Hideo D. Takagi

**Affiliations:** a Department of Chemistry, Okayama University, Okayama 700-8530, Japan; b Graduate School of Science and Research Center for Material Science, Nagoya, University, Chikusa, Nagoya 464-8602, Japan

**Keywords:** crystal structure, 8-quinolylphos­phane, stacking inter­action, geometrical structure, *trans* influence

## Abstract

The crystal structures of di­chlorido­palladium(II), -platinum(II) and -rhodium(III) complexes containing 8-(di­phenyl­phosphan­yl)quinoline, (*SP*-4)-[PdCl_2_(C_21_H_16_NP)], (1) [systematic name: di­chlor­ido­(8-di­phenyl­phosphanyl­quinoline)­palladium(II)], (*SP*-4)-[PtCl_2_(C_21_H_16_NP)]·CH_2_Cl_2_, (2) [systematic name: di­chlorido­(8-di­phenyl­phos­phanyl­quinoline)­platinum(II) dichlorometh­ane monosolvate], and (*OC*-6–32)-[RhCl_2_(C_21_H_16_NP)_2_]PF_6_·0.5CH_2_Cl_2_·0.5CH_3_OH, (3) [systematic name: *cis*-di­chlor­ido­bis­(8-di­phenyl­phosphanyl­quinoline)­rhodium(III) hexa­fluorido­phos­phate di­chloro­methane/­methanol hemisolvate] are reported. In these complexes, the phosphanyl­quinoline acts as a bidentate ligand, forming a planar asymmetrical five-membered chelate ring. The palladium(II) and platinum(II) complex mol­ecules in (1) and (2), respectively, show a typical square-planar coordination geometry and form a dimeric structure through an inter­molecular π–π stacking inter­action between the quinolyl rings. The centroid–centroid distances between the stacked six-membered rings in (1) and (2) are 3.633 (2) and 3.644 (2) Å, respectively. The cationic rhodium(III) complex in (3) has a *cis(Cl),cis(P),cis(N)* (*OC*-6–32) configuration of the ligands, in which two kinds of intra­molecular π–π stacking inter­actions are observed: between the quinolyl and phenyl rings and between two phenyl rings, the centroid–centroid distances being 3.458 (2) and 3.717 (2) Å, respectively. The PF_6_^−^ anion in (3) is rotationally disordered, the site occupancies of each F atom being 0.613 (14) and 0.387 (14). The CH_2_Cl_2_ and CH_3_OH solvent mol­ecules are also disordered and equal site occupancies of 0.5 are assumed.

## Abstract 2

8-(Di­phenyl­phosphan­yl)quinoline (Ph_2_Pqn) acts as an asymmetric bidentate ligand to form a planar five-membered chelate ring in the di­chlorido­palladium(II) and -platinum(II) complexes, (*SP*-4)-[*M*Cl_2_(Ph_2_Pqn)] (*M* = Pd or Pt), as well as in the di­chlorido­rhodium(III) complex, (*OC*-6-32)-[RhCl_2_(Ph_2_Pqn)_2_]PF_6_.

## Chemical context   

8-Quinolylphosphanes are an inter­esting class of ligands because they form a planar asymmetrical five-membered chelate ring *via* coordination through quinoline-*N* and phosphane-*P* atoms (Issleib & Hörnig, 1972 ▸; Salem & Wild, 1992 ▸; Wehman *et al.*, 1997 ▸). The electronic differentiation of the donor groups, in particular their π-bonding natures, may stabilize unusual electronic states of their transition metal complexes (Espinet & Soulantica, 1999 ▸). In addition, the steric requirement from the quinolyl moiety often has a strong influence on the properties of their metal complexes. For example, the nickel(II) and palladium(II) complexes containing two 8-(di­phenyl­phosphan­yl)quinoline (Ph_2_Pqn) mol­ecules with a *cis(P)* configuration showed a severe distortion of the square-planar coordination geometry around Ni^II^ and Pd^II^ as a result of the steric hindrance between mutually *cis*-positioned quinolyl groups (Suzuki, 2004 ▸; Hashimoto *et al.*, 2010 ▸). Several crystallographic studies have been performed for other Ph_2_Pqn complexes, as described in §4, but not for the platinum(II) and rhodium(III) complexes. In 1979, the preparation and spectroscopic characterization of [*M*Cl_2_(Ph_2_Pqn)] (*M* = Pd^II^, Pt^II^, and Rh^II^) was reported (Hudali *et al.*, 1979 ▸), but the crystal structures of these complexes were not confirmed, except for [PdCl_2_(Ph_2_Pqn)]·CH_2_Cl_2_ (Bastanov *et al.*, 2009 ▸). In particular, it is worthwhile to reinvestigate the rhodium(II) complex because it was prepared from RhCl_3_·3H_2_O and Ph_2_Pqn in acetone (Hudali *et al.*, 1979 ▸).
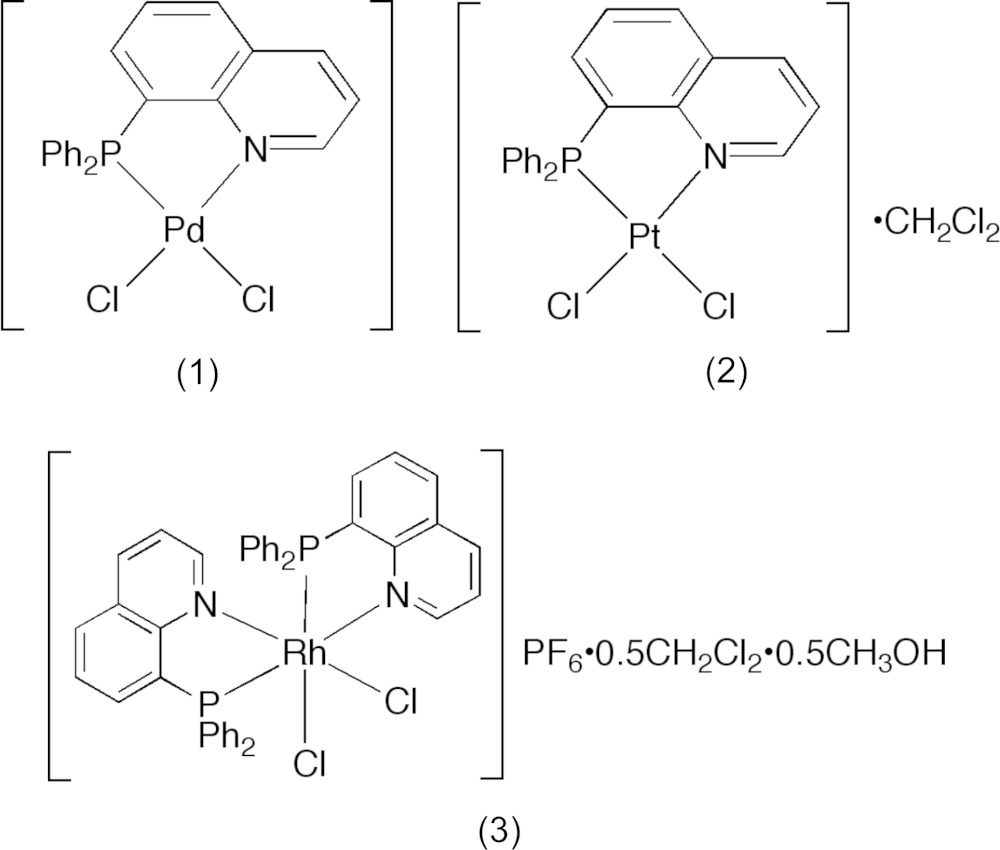


## Structural commentary   

**Figure 1:**
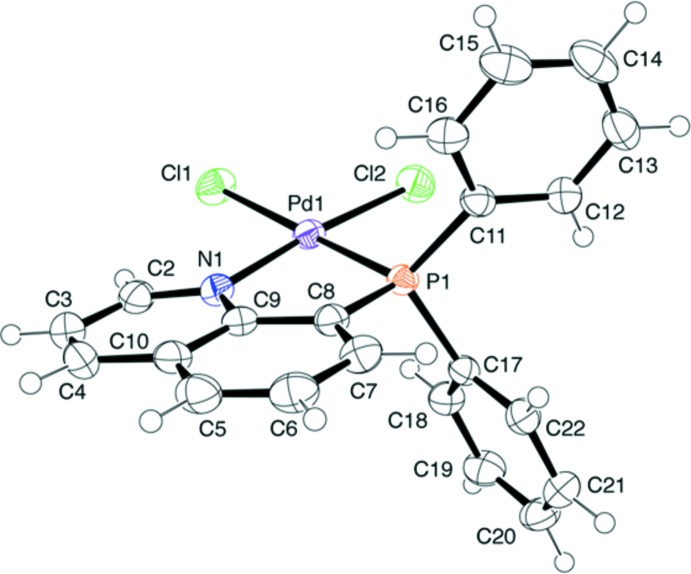
An *ORTEP* of the mol­ecular structure of [PdCl_2_(Ph_2_Pqn)], (1), showing the atom-numbering scheme, with displacement ellipsoids drawn at the 50% probability level.

A yellow block-shaped crystal of the Pd^II^ complex, (*SP*-4)-[PdCl_2_(Ph_2_Pqn)], (1), recrystallized from hot aceto­nitrile, was used for the X-ray diffraction analysis. The complex mol­ecule (Fig. 1 ▸) has a typical square-planar coordination geometry with a chelating Ph_2_Pqn ligand, whose P1—Pd1—N1 bite angle is 84.75 (6)°. The quinolyl plane is almost co-planar to the Pd^II^ coordination plane; the dihedral angle between these planes is only 8.58 (3)°. The two Pd—Cl bonds show a significant difference in length [Pd1—Cl1 2.3716 (6) *vs* Pd1—Cl2 2.2885 (7) Å], indicating a strong *trans* influence of the phosphane donor group. The corresponding Pd—Cl bond in *cis(P)*-[PdCl(Ph_2_Pqn)_2_]BF_4_, which is also *trans* to the phosphane donor of Ph_2_Pqn, was similarly long at 2.375 (2) Å (Suzuki, 2004 ▸). On the other hand, the Pd1—P1 bond [2.2026 (6) Å] in (1) is slightly shorter than those in *cis(P)*-[PdCl(Ph_2_Pqn)_2_]BF_4_ and *cis(P)*-[Pd(Ph_2_Pqn)_2_]*X*_2_ (*X* = Cl or Br) [2.229 (2)–2.267 (2) Å], presumably due to the steric congestion in the above bis­(Ph_2_Pqn)-type complexes. The Pd1—N1 bond length in (1) is 2.065 (2) Å. The dihedral angles between the quinolyl ring system and the two phenyl rings of the coordinated Ph_2_Pqn are 72.34 (8) and 74.79 (8)°.

**Figure 2:**
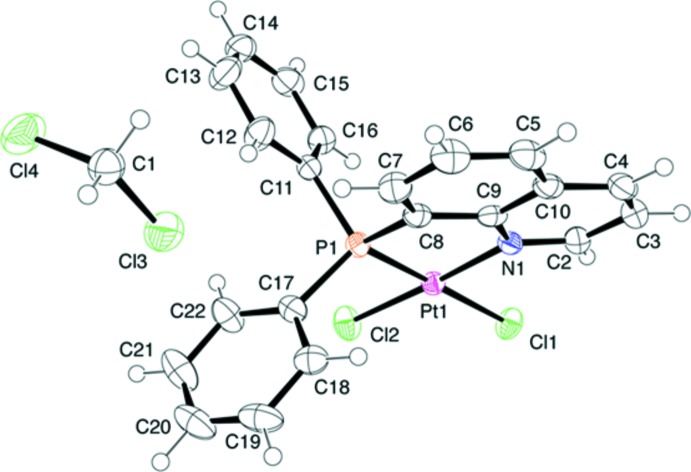
An *ORTEP* of the mol­ecular structure of [PtCl_2_(Ph_2_Pqn)]·CH_2_Cl_2_, (2), showing the atom-numbering scheme, with displacement ellipsoids drawn at the 50% probability level.

When the platinum(II) complex was recrystallized from di­chloro­methane, the resulting crystals contained a CH_2_Cl_2_ mol­ecule per a complex mol­ecule: [PtCl_2_(Ph_2_Pqn)]·CH_2_Cl_2_ (2). The X-ray analysis revealed that it was isomorphous with the Pd^II^ analogue, [PdCl_2_(Ph_2_Pqn)]·CH_2_Cl_2_, which has been deposited in the Cambridge Structural Database (Bastanov *et al.*, 2009 ▸). The mol­ecular structure of the Pt^II^ complex moiety with a square-planar coordination geometry (Fig. 2 ▸) is very similar to the above Pd^II^ complex in (1). The Pt1—P1 and Pt1—N1 bond lengths are 2.1963 (6) and 2.051 (2) Å, respectively, and the Ph_2_Pqn bite angle (P1—Pt1—N1) is 85.44 (6)°. The Pt1—Cl1 and Pt1—Cl2 bond lengths are 2.3747 (6) and 2.3002 (7) Å, respectively, also indicative of a strong *trans* influence of the phosphane donor group.

**Figure 3:**
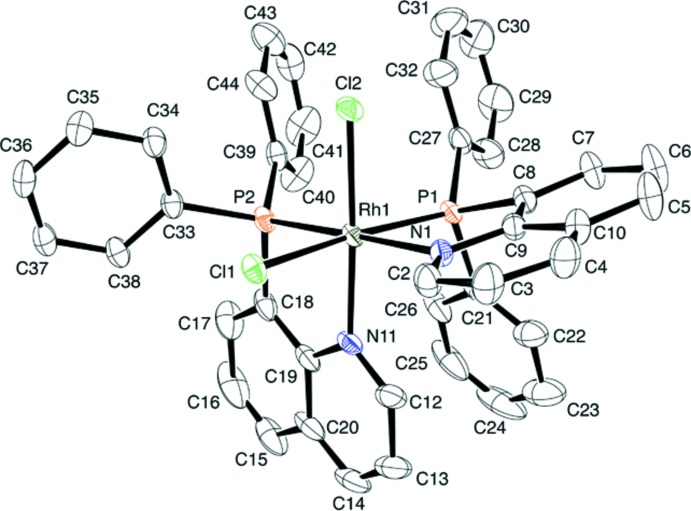
An *ORTEP* of the complex molecule in (*OC*-6–32)-[RhCl_2_(Ph_2_Pqn)_2_]PF_6_·0.5CH_2_Cl_2_·0.5CH_3_OH, (3), showing the atom-numbering scheme, with displacement ellipsoids drawn at 30% probability level. Hydrogen atoms are omitted for clarity.

Pale yellow prismatic crystals of [RhCl_2_(Ph_2_Pqn)_2_]PF_6_·0.5CH_2_Cl_2_·0.5CH_3_OH (3) were analyzed by the X-ray diffraction method, and it was revealed that the complex cation has an octa­hedral coordination geometry with a *cis(Cl),cis(P),cis(N)* (*OC*-6-32) configuration (Figs. 3 ▸ and 4 ▸). As a result of the strong *trans* influence of the phosphane donor, the two Rh—Cl and the two Rh—N bond lengths are significantly different from each other. The Rh1—Cl1 bond [2.3787 (6) Å] is longer by 0.045 Å than Rh1—Cl2 [2.3338 (7) Å], while Rh1—N1 [2.168 (2) Å] is longer by 0.10 Å than Rh1—N11 [2.065 (2) Å]. This fact suggests that the *trans* influence of the phosphane donor is much effective for the Rh—N(quinoline) bond rather than the Rh—Cl bond. Two slightly deviated Rh—P bond lengths [Rh1—P1 2.2897 (7) *vs*. Rh1—P2 2.2531 (8) Å] seem to result from different steric congestion around the P donor atoms. The larger bond angle of P1—Rh1—P2 [100.55 (3)°] than the ideal right angle is also suggestive of steric inter­action between the two phosphane groups. However, the mol­ecular structure of the complex cation (Fig. 3 ▸) also suggests an intra­molecular π–π stacking inter­action between the C27–C32 and C39–C44 phenyl rings. The centroid–centroid distance between these rings is 3.717 (2) Å. An other intra­molecular π–π stacking inter­action is also found between the N11/C12–C14/C20/C19 ring of the quinolyl substituent and the C21–C26 phenyl ring, the centroid–centroid distance being 3.458 (2) Å. These inter­actions could stabilize the *cis(Cl),cis(P),cis(N)* configuration of the Rh^III^ complex cation [RhCl_2_(Ph_2_Pqn)_2_]^+^.

**Figure 4:**
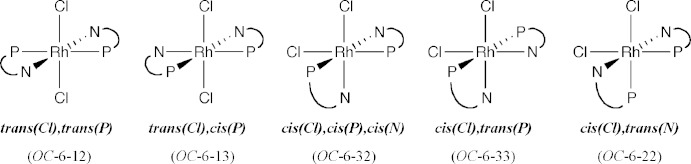
Possible configurations and notation for the [RhCl_2_(P–N)_2_]^+^ complex cation.

The crystal structures of the related complexes with (2-amino­eth­yl)di­phenyl­phosphane, [RhCl_2_(Ph_2_PCH_2_CH_2_NH_2_)_2_]^+^, were reported to have the *trans(Cl),cis(P)* (*OC*-6-13) or *cis(Cl),trans(P)* (*OC*-6-33) configuration (Fig. 4 ▸) (Galsbøl *et al.*, 1986 ▸). If the *trans(Cl),cis(P)* configuration were assumed for the present Ph_2_Pqn complex, the mol­ecule would have severe steric hindrance between the *ortho*-H atoms of the mutually *cis*-positioned quinolyl groups, as observed in the crystal structures of *cis(P)*-[Pd(Ph_2_Pqn)_2_]*X*_2_ (Suzuki, 2004 ▸). The *trans(P)* configurations, *i.e.*, *trans(Cl),trans(P)* (*OC*-6-12) and *cis(Cl),trans(P)* (*OC*-6-33), would be unfavorable due to the mutually *trans* disposition of the phosphane groups having a strong *trans* influence. The last configuration, *cis(Cl),trans(N)* (*OC*-6-22), cannot form an intra­molecular stacking inter­action between the aryl groups of the phosphanes. Therefore, the observed *cis(Cl),cis(P),cis(N)* geometrical isomer could be the most favorable from the steric and electronic points of views.

## Supra­molecular features   

**Figure 5:**
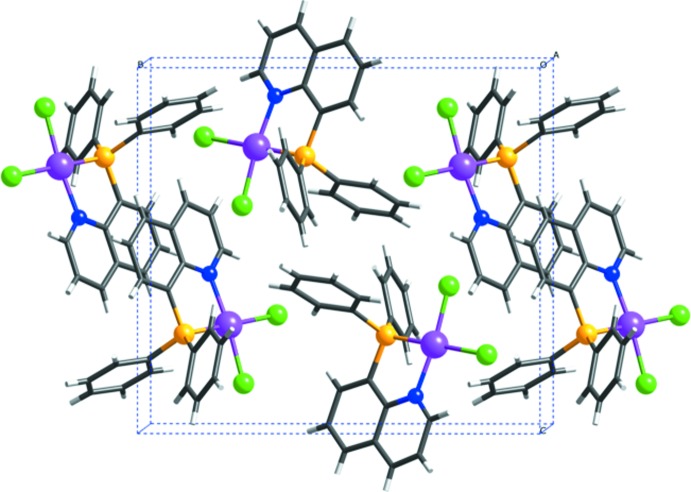
A view of the crystal packing of [PdCl_2_(Ph_2_Pqn)], (1), illustrating the π–π stacking inter­actions between the complexes. Color code: Pd, purple; Cl, green; P, yellow; N, blue; C, black; and H, gray.

In the crystal structure of (1), there is an inter­molecular π–π stacking inter­action between the quinolyl planes, forming an inversion dimer (Fig. 5 ▸). The centroid–centroid distance between the N1/C2–C4/C10/C9 ring and the C5^i^–C10^i^ ring of the neighbouring mol­ecule [symmetry code: (i) 1 – *x*, 1 – *y*, 2 – *z*] is 3.633 (2) Å.

**Figure 6:**
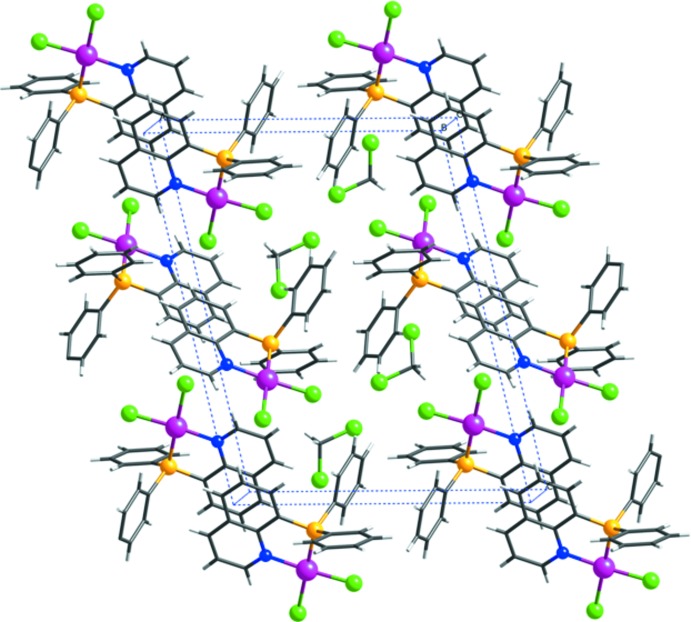
A view of the crystal packing of [PtCl_2_(Ph_2_Pqn)]·CH_2_Cl_2_, (2), illustrating the π–π stacking inter­actions between the complexes. Color code: Pt, purple; Cl, green; P, yellow; N, blue; C, black; and H, gray.

The Pt^II^ complex in (2) also forms an inversion dimer unit by an inter­molecular π–π stacking inter­action between the quinolyl rings of neighbouring mol­ecules (Fig. 6 ▸). The centroid–centroid distance between the N1/C2–C4/C10/C9 ring and the C5^ii^–C10^ii^ ring of the neighbouring mol­ecule [symmetry code: (ii) 1 – *x*, –*y*, 1 – *z*] is 3.644 (2) Å.

No remarkable inter­molecular stacking or hydrogen-bonding inter­actions are observed in the crystal structure of (3).

## Database survey   

The crystal structure of Ph_2_Pqn was reported previously (Nag *et al.*, 2010 ▸). Several metal complexes containing Ph_2_Pqn have also reported by us and others, *e.g.*, [Ni(Ph_2_Pqn)_2_](BF_4_)_*n*_ (*n* = 1 or 2; Hashimoto *et al.*, 2010 ▸), [Pd(Ph_2_Pqn)_2_]*X*_2_ (*X* = Cl, Br, or BF_4_; Suzuki, 2004 ▸), [Ru(bpy)_2_(Ph_2_Pqn)](PF_6_)_2_ (bpy = 2,2-bi­pyridine; Suzuki *et al.*, 2002 ▸), [Cp*Ir(N_3_)(Ph_2_Pqn)] (Cp* = penta­methyl­cyclo­penta­dienyl; Suzuki *et al.*, 2009 ▸), [Cu(Ph_2_Pqn)_2_]BF_4_ (Suzuki *et al.*, 2011 ▸), [NiCl(C_10_H_7_)(Ph_2_Pqn)] (C_10_H_7_ = 1-naphthyl; Sun *et al.*, 2002 ▸), [Cu(Ph_2_Pqn)_2_]PF_6_ and [Zn*X*_2_(Ph_2_Pqn)] (*X* = Cl, Br, or I; Tsukuda *et al.*, 2009 ▸), [Cu(Ph_2_Pqn){(Ph_2_PC_6_H_4_)_2_O}]BF_4_ (Qin *et al.*, 2009 ▸), [AuCl(Ph_2_Pqn)] (Monkowius *et al.*, 2009 ▸), [PdCl(C_3_H_5_)(Ph_2_Pqn)], [Pd(C_3_H_5_)(Ph_2_Pqn)]ClO_4_, [Pd(Ph_2_Pqn)(MeOOCC≡CCOOMe)] and [Pd(Ph_2_Pqn){MeOOC(Me)C=CCOOMe}] (C_3_H_5_ = allyl; Canovese *et al.*, 2010 ▸). In addition, the crystal structure of [PdCl_2_(Ph_2_Pqn)]·CH_2_Cl_2_ has been deposited (Bastanov *et al.*, 2009 ▸).

## Synthesis and crystallization   

The ligand, Ph_2_Pqn, was prepared according to a literature method (Feltham & Metzger, 1971 ▸; Aguirre *et al.*, 2007 ▸). The di­chlorido­palladium(II) and platinum(II) complexes, [PdCl_2_(Ph_2_Pqn)] and [PtCl_2_(Ph_2_Pqn)], were prepared by the method reported previously by Hudali *et al.* (1979 ▸). The palladium(II) complex was recrystallized from hot aceto­nitrile to afford yellow block-shaped crystals of [PdCl_2_(Ph_2_Pqn)], (1). Analysis calculated for C_21_H_16_Cl_2_NPPd: C 51.4, H 3.29, N 2.85%. Found: C 51.2, H 3.25, N 2.87%.

The colorless platelet crystals of the platinum(II) complex, [PtCl_2_(Ph_2_Pqn)]·CH_2_Cl_2_, (2), were obtained by recrystallization from di­chloro­methane. Analysis calculated for C_21_H_16_Cl_2_NPPt: C 43.5, H 2.78, N 2.42%. Found (after drying completely): C 42.8, H 2.75, N 2.44%.

The PF_6_ salt of the di­chlorido­rhodium(III) complex, [RhCl_2_(Ph_2_Pqn)_2_]PF_6_, was precipitated from a methanol solution of RhCl_3_(Ph_2_Pqn)_2_(H_2_O), which was prepared by a reaction of RhCl_3_·3H_2_O and two equivalent amounts of Ph_2_Pqn in boiling water, by addition of a saturated methanol solution of NH_4_PF_6_. The crude product was recrystallized from a mixture of di­chloro­methane and methanol, affording pale-yellow prismatic crystals of [RhCl_2_(Ph_2_Pqn)_2_]PF_6_·0.5CH_2_Cl_2_·0.5CH_3_OH (3). These crystals were efflorescent when they were picked up from the mother liquor. Analysis calculated for C_42_H_32_Cl_2_F_6_N_2_P_3_Rh·2H_2_O: C 51.4, H 3.70, N 2.85%. Found (after drying completely): C 51.6, H 3.55, N 2.85%.

## Refinement   

Crystal data, data collection and structure refinement details are summarized in Table 1 ▸. All H atoms were refined using a riding model, with O—H = 0.84 Å and C—H = 0.95 (aromatic), 0.99 (methyl­ene) or 0.98 (meth­yl) Å, and with *U*_iso_(H) = 1.2 or 1.5*U*_eq_(C,O). During the refinement for (3), each F atom of the PF_6_^−^ anion was found to have a large displacement ellipsoid elongated in the direction perpendic­ular to the P—F bond, which was attributable to rotational disorder of the anion over two positions. The occupancies of each F atom refined to 0.613 (14) and 0.387 (14). In addition, since the crystal structure of (3) contains a void accessible for a solvent mol­ecule, disordered CH_2_Cl_2_ and CH_3_OH mol­ecules with equal probabilities of 0.5 were assumed. In the refinement, the P—F, C—Cl and C—O bond lengths and the Cl—C—Cl bond angle were restrained to be 1.55 (1), 1.75 (1), 1.42 (2) Å and 112.0 (2)°, respectively. Rigid bond restraints were also applied for the disordered CH_2_Cl_2_ and CH_3_OH mol­ecules.

## Supplementary Material

Crystal structure: contains datablock(s) 1, 2, 3. DOI: 10.1107/S2056989015006076/is5392sup1.cif

Structure factors: contains datablock(s) 1. DOI: 10.1107/S2056989015006076/is53921sup2.hkl

Structure factors: contains datablock(s) 2. DOI: 10.1107/S2056989015006076/is53922sup3.hkl

Structure factors: contains datablock(s) 3. DOI: 10.1107/S2056989015006076/is53923sup4.hkl

CCDC references: 1056041, 1056040, 1056039

Additional supporting information:  crystallographic information; 3D view; checkCIF report

## Figures and Tables

**Table 1:** Experimental details

	(1)	(2)	(3)
Crystal data			
Chemical formula	[PdCl_2_(C_21_H_16_NP)]	[PtCl_2_(C_21_H_16_NP)]·CH_2_Cl_2_	[RhCl_2_(C_21_H_16_NP)_2_](PF_6_)·0.5CH_2_Cl_2_·0.5CH_4_O
*M*_r_	490.62	664.23	1003.90
Crystal system, space group	Monoclinic, *P*2_1_/*n*	Monoclinic, *P*2_1_/*c*	Triclinic, *P* 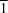
Temperature (K)	200	200	200
*a*, *b*, *c* (Å)	9.0293 (5), 15.2154 (8), 13.7936 (6)	13.9280 (5), 9.2371 (3), 17.8941 (6)	9.841 (5), 13.825 (6), 16.167 (8)
α, β, γ (°)	90, 91.8197 (13), 90	90, 102.8447 (10), 90	87.307 (19), 81.80 (2), 70.819 (18)
*V* (Å^3^)	1894.07 (16)	2244.53 (13)	2056.2 (17)
*Z*	4	4	2
Radiation type	Mo *K*α	Mo *K*α	Mo *K*α
μ (mm^−1^)	1.35	6.81	0.79
Crystal size (mm)	0.18 × 0.15 × 0.12	0.25 × 0.24 × 0.05	0.20 × 0.20 × 0.15

Data collection			
Diffractometer	Rigaku R-AXIS RAPID	Rigaku R-AXIS RAPID	Rigaku R-AXIS RAPID
Absorption correction	Numerical (*NUMABS*; Rigaku, 1999 ▸)	Numerical (*NUMABS*; Rigaku, 1999 ▸)	Multi-scan (*ABSCOR.*; Rigaku, 1995 ▸)
*T*_min_, *T*_max_	0.693, 0.850	0.281, 0.727	0.848, 0.882
No. of measured, independent and observed [*I* > 2σ(*I*)] reflections	18103, 4283, 3913	20995, 5081, 4665	20385, 9338, 7245
*R*_int_	0.034	0.028	0.046
(sin θ/λ)_max_ (Å^−1^)	0.649	0.648	0.649

Refinement			
*R*[*F* ^2^ > 2σ(*F* ^2^)], *wR*(*F* ^2^), *S*	0.028, 0.074, 0.92	0.017, 0.037, 0.98	0.044, 0.120, 1.06
No. of reflections	4283	5081	9338
No. of parameters	235	262	605
No. of restraints	0	0	20
H-atom treatment	H-atom parameters constrained	H-atom parameters constrained	H-atom parameters constrained
Δρ_max_, Δρ_min_ (e Å^−3^)	0.84, −0.50	0.58, −0.60	0.68, −1.03

Computer programs: *PROCESS-AUTO* (Rigaku, 1998 ▸), *CrystalStructure* (Rigaku, 2010 ▸), *DIRDIF99-PATTY* (Beurskens *et al.*, 1999 ▸), *SHELXS2013* (Sheldrick, 2008 ▸), *SHELXL2013* (Sheldrick, 2015 ▸) and *ORTEP-3 for Windows* (Farrugia, 2012 ▸).

## supplementary crystallographic information

### (1) Dichlorido(8-diphenylphosphanylquinoline)palladium(II) Crystal data

**Table:**

[PdCl_2_(C_21_H_16_NP)]	*F*(000) = 976
*M**_r_* = 490.62	*D*_x_ = 1.721 Mg m^−^^3^
Monoclinic, *P*2_1_/*n*	Mo *K*α radiation, λ = 0.71075 Å
*a* = 9.0293 (5) Å	Cell parameters from 10456 reflections
*b* = 15.2154 (8) Å	θ = 3.0–27.5°
*c* = 13.7936 (6) Å	µ = 1.35 mm^−^^1^
β = 91.8197 (13)°	*T* = 200 K
*V* = 1894.07 (16) Å^3^	Block, yellow
*Z* = 4	0.18 × 0.15 × 0.12 mm

### (1) Dichlorido(8-diphenylphosphanylquinoline)palladium(II) Data collection

**Table:**

Rigaku R-AXIS RAPID diffractometer	4283 independent reflections
Radiation source: fine-focus sealed tube	3913 reflections with *I* > 2σ(*I*)
Detector resolution: 10.00 pixels mm^-1^	*R*_int_ = 0.034
ω scans	θ_max_ = 27.5°, θ_min_ = 3.1°
Absorption correction: numerical (*NUMABS*; Rigaku, 1999)	*h* = −11→9
*T*_min_ = 0.693, *T*_max_ = 0.850	*k* = −19→19
18103 measured reflections	*l* = −17→17

### (1) Dichlorido(8-diphenylphosphanylquinoline)palladium(II) Refinement

**Table:**

Refinement on *F*^2^	Primary atom site location: structure-invariant direct methods
Least-squares matrix: full	Secondary atom site location: difference Fourier map
*R*[*F*^2^ > 2σ(*F*^2^)] = 0.028	Hydrogen site location: inferred from neighbouring sites
*wR*(*F*^2^) = 0.074	H-atom parameters constrained
*S* = 0.92	*w* = 1/[σ^2^(*F*_o_^2^) + (0.0416*P*)^2^ + 2.3772*P*] where *P* = (*F*_o_^2^ + 2*F*_c_^2^)/3
4283 reflections	(Δ/σ)_max_ = 0.001
235 parameters	Δρ_max_ = 0.84 e Å^−^^3^
0 restraints	Δρ_min_ = −0.50 e Å^−^^3^

### (1) Dichlorido(8-diphenylphosphanylquinoline)palladium(II) Special details

**Table:**

Geometry. All e.s.d.'s (except the e.s.d. in the dihedral angle between two l.s. planes) are estimated using the full covariance matrix. The cell e.s.d.'s are taken into account individually in the estimation of e.s.d.'s in distances, angles and torsion angles; correlations between e.s.d.'s in cell parameters are only used when they are defined by crystal symmetry. An approximate (isotropic) treatment of cell e.s.d.'s is used for estimating e.s.d.'s involving l.s. planes.

### (1) Dichlorido(8-diphenylphosphanylquinoline)palladium(II) Fractional atomic coordinates and isotropic or equivalent isotropic displacement parameters (Å^2^)

**Table:**

	*x*	*y*	*z*	*U*_iso_*/*U*_eq_	
Pd1	0.42495 (2)	0.29307 (2)	0.78114 (2)	0.02590 (7)	
Cl1	0.57514 (7)	0.16783 (4)	0.81289 (5)	0.04011 (15)	
Cl2	0.39715 (8)	0.25626 (5)	0.62073 (5)	0.04049 (16)	
P1	0.28188 (6)	0.40803 (4)	0.75258 (4)	0.02469 (13)	
N1	0.4378 (2)	0.33998 (13)	0.92166 (15)	0.0296 (4)	
C2	0.5290 (3)	0.30655 (17)	0.9896 (2)	0.0359 (6)	
H2	0.5960	0.2618	0.9714	0.043*	
C3	0.5315 (3)	0.33376 (19)	1.0863 (2)	0.0414 (6)	
H3	0.5995	0.3081	1.1320	0.050*	
C4	0.4363 (3)	0.39706 (19)	1.11443 (19)	0.0400 (6)	
H4	0.4334	0.4139	1.1807	0.048*	
C5	0.2453 (3)	0.50817 (18)	1.06576 (19)	0.0396 (6)	
H5	0.2397	0.5283	1.1307	0.047*	
C6	0.1606 (3)	0.54738 (18)	0.9943 (2)	0.0391 (6)	
H6	0.0978	0.5950	1.0101	0.047*	
C7	0.1650 (3)	0.51819 (17)	0.89779 (18)	0.0333 (5)	
H7	0.1053	0.5460	0.8489	0.040*	
C8	0.2560 (3)	0.44917 (15)	0.87408 (17)	0.0271 (5)	
C9	0.3462 (3)	0.40817 (15)	0.94699 (17)	0.0280 (5)	
C10	0.3411 (3)	0.43799 (17)	1.04423 (18)	0.0346 (5)	
C11	0.0989 (3)	0.39004 (15)	0.70011 (17)	0.0279 (5)	
C12	0.0674 (3)	0.40697 (17)	0.60209 (19)	0.0335 (5)	
H12	0.1406	0.4325	0.5629	0.040*	
C13	−0.0709 (3)	0.38636 (19)	0.5624 (2)	0.0402 (6)	
H13	−0.0932	0.3987	0.4960	0.048*	
C14	−0.1766 (3)	0.3481 (2)	0.6186 (2)	0.0440 (7)	
H14	−0.2709	0.3336	0.5905	0.053*	
C15	−0.1463 (3)	0.3307 (2)	0.7151 (2)	0.0432 (7)	
H15	−0.2198	0.3042	0.7533	0.052*	
C16	−0.0090 (3)	0.35166 (17)	0.75691 (19)	0.0341 (5)	
H16	0.0114	0.3400	0.8237	0.041*	
C17	0.3685 (3)	0.49474 (15)	0.68551 (16)	0.0260 (5)	
C18	0.5132 (3)	0.48296 (17)	0.65615 (18)	0.0325 (5)	
H18	0.5608	0.4277	0.6651	0.039*	
C19	0.5880 (3)	0.55202 (19)	0.61371 (19)	0.0391 (6)	
H19	0.6871	0.5441	0.5943	0.047*	
C20	0.5192 (3)	0.63163 (19)	0.59974 (19)	0.0405 (6)	
H20	0.5711	0.6788	0.5711	0.049*	
C21	0.3749 (3)	0.64331 (18)	0.6273 (2)	0.0393 (6)	
H21	0.3273	0.6983	0.6163	0.047*	
C22	0.2985 (3)	0.57554 (17)	0.67083 (18)	0.0333 (5)	
H22	0.1996	0.5841	0.6904	0.040*	

### (1) Dichlorido(8-diphenylphosphanylquinoline)palladium(II) Atomic displacement parameters (Å^2^)

**Table:**

	*U*^11^	*U*^22^	*U*^33^	*U*^12^	*U*^13^	*U*^23^
Pd1	0.02371 (11)	0.02217 (11)	0.03178 (12)	−0.00047 (6)	0.00035 (7)	0.00150 (6)
Cl1	0.0335 (3)	0.0306 (3)	0.0562 (4)	0.0070 (3)	0.0014 (3)	0.0060 (3)
Cl2	0.0487 (4)	0.0367 (3)	0.0361 (3)	0.0004 (3)	0.0012 (3)	−0.0069 (2)
P1	0.0232 (3)	0.0233 (3)	0.0276 (3)	−0.0006 (2)	0.0002 (2)	0.0007 (2)
N1	0.0268 (10)	0.0295 (11)	0.0323 (11)	−0.0045 (8)	−0.0015 (8)	0.0046 (8)
C2	0.0336 (14)	0.0307 (13)	0.0427 (15)	−0.0045 (10)	−0.0076 (11)	0.0079 (10)
C3	0.0452 (16)	0.0406 (15)	0.0376 (15)	−0.0086 (13)	−0.0108 (11)	0.0079 (11)
C4	0.0478 (17)	0.0442 (16)	0.0276 (13)	−0.0135 (13)	−0.0061 (11)	0.0016 (11)
C5	0.0490 (17)	0.0379 (14)	0.0322 (14)	−0.0090 (12)	0.0081 (11)	−0.0077 (10)
C6	0.0426 (16)	0.0317 (14)	0.0436 (16)	−0.0012 (11)	0.0109 (11)	−0.0056 (11)
C7	0.0337 (13)	0.0300 (13)	0.0366 (14)	−0.0012 (10)	0.0057 (10)	−0.0008 (10)
C8	0.0260 (12)	0.0269 (12)	0.0286 (12)	−0.0045 (9)	0.0020 (9)	0.0000 (9)
C9	0.0273 (12)	0.0252 (11)	0.0314 (12)	−0.0074 (9)	0.0006 (9)	0.0010 (9)
C10	0.0397 (15)	0.0321 (13)	0.0320 (13)	−0.0117 (11)	0.0001 (10)	0.0006 (10)
C11	0.0247 (12)	0.0237 (11)	0.0354 (13)	0.0009 (9)	−0.0006 (9)	−0.0019 (9)
C12	0.0334 (13)	0.0317 (13)	0.0351 (14)	−0.0009 (10)	−0.0032 (10)	0.0010 (10)
C13	0.0360 (15)	0.0385 (15)	0.0451 (16)	0.0083 (11)	−0.0136 (11)	−0.0063 (11)
C14	0.0280 (14)	0.0427 (16)	0.0607 (19)	0.0062 (12)	−0.0074 (12)	−0.0177 (13)
C15	0.0275 (14)	0.0441 (16)	0.0583 (19)	−0.0037 (12)	0.0083 (12)	−0.0111 (13)
C16	0.0296 (13)	0.0351 (13)	0.0375 (14)	−0.0036 (10)	0.0023 (10)	−0.0022 (10)
C17	0.0257 (12)	0.0273 (11)	0.0250 (11)	−0.0040 (9)	−0.0015 (8)	0.0006 (8)
C18	0.0302 (13)	0.0298 (13)	0.0375 (14)	0.0002 (10)	0.0030 (10)	−0.0015 (10)
C19	0.0343 (14)	0.0434 (15)	0.0400 (15)	−0.0089 (12)	0.0096 (11)	−0.0019 (11)
C20	0.0496 (17)	0.0358 (14)	0.0362 (14)	−0.0142 (12)	0.0028 (11)	0.0044 (11)
C21	0.0473 (16)	0.0287 (13)	0.0415 (15)	−0.0018 (11)	−0.0053 (11)	0.0064 (10)
C22	0.0295 (13)	0.0328 (13)	0.0374 (14)	0.0000 (10)	−0.0013 (10)	0.0033 (10)

### (1) Dichlorido(8-diphenylphosphanylquinoline)palladium(II) Geometric parameters (Å, º)

**Table:**

Pd1—N1	2.065 (2)	C9—C10	1.418 (3)
Pd1—P1	2.2026 (6)	C11—C12	1.397 (4)
Pd1—Cl2	2.2885 (7)	C11—C16	1.397 (3)
Pd1—Cl1	2.3716 (6)	C12—C13	1.384 (4)
P1—C11	1.804 (2)	C12—H12	0.9500
P1—C17	1.804 (2)	C13—C14	1.377 (4)
P1—C8	1.811 (2)	C13—H13	0.9500
N1—C2	1.329 (3)	C14—C15	1.377 (4)
N1—C9	1.379 (3)	C14—H14	0.9500
C2—C3	1.396 (4)	C15—C16	1.387 (4)
C2—H2	0.9500	C15—H15	0.9500
C3—C4	1.356 (4)	C16—H16	0.9500
C3—H3	0.9500	C17—C22	1.394 (3)
C4—C10	1.418 (4)	C17—C18	1.392 (3)
C4—H4	0.9500	C18—C19	1.388 (4)
C5—C6	1.365 (4)	C18—H18	0.9500
C5—C10	1.412 (4)	C19—C20	1.372 (4)
C5—H5	0.9500	C19—H19	0.9500
C6—C7	1.405 (4)	C20—C21	1.381 (4)
C6—H6	0.9500	C20—H20	0.9500
C7—C8	1.379 (3)	C21—C22	1.388 (4)
C7—H7	0.9500	C21—H21	0.9500
C8—C9	1.418 (3)	C22—H22	0.9500
			
N1—Pd1—P1	84.75 (6)	C5—C10—C9	118.6 (2)
N1—Pd1—Cl2	173.28 (6)	C5—C10—C4	123.4 (2)
P1—Pd1—Cl2	88.61 (2)	C9—C10—C4	117.9 (3)
N1—Pd1—Cl1	95.14 (6)	C12—C11—C16	119.7 (2)
P1—Pd1—Cl1	178.94 (2)	C12—C11—P1	121.08 (19)
Cl2—Pd1—Cl1	91.51 (3)	C16—C11—P1	119.00 (19)
C11—P1—C17	108.18 (11)	C13—C12—C11	119.7 (3)
C11—P1—C8	106.28 (11)	C13—C12—H12	120.2
C17—P1—C8	107.01 (11)	C11—C12—H12	120.2
C11—P1—Pd1	118.41 (8)	C12—C13—C14	120.4 (3)
C17—P1—Pd1	114.27 (8)	C12—C13—H13	119.8
C8—P1—Pd1	101.60 (8)	C14—C13—H13	119.8
C2—N1—C9	118.2 (2)	C15—C14—C13	120.4 (3)
C2—N1—Pd1	122.99 (19)	C15—C14—H14	119.8
C9—N1—Pd1	118.79 (16)	C13—C14—H14	119.8
N1—C2—C3	123.4 (3)	C14—C15—C16	120.3 (3)
N1—C2—H2	118.3	C14—C15—H15	119.8
C3—C2—H2	118.3	C16—C15—H15	119.8
C4—C3—C2	119.5 (3)	C15—C16—C11	119.5 (3)
C4—C3—H3	120.2	C15—C16—H16	120.2
C2—C3—H3	120.2	C11—C16—H16	120.2
C3—C4—C10	119.5 (3)	C22—C17—C18	119.8 (2)
C3—C4—H4	120.3	C22—C17—P1	121.15 (18)
C10—C4—H4	120.3	C18—C17—P1	118.78 (19)
C6—C5—C10	120.8 (2)	C19—C18—C17	119.9 (2)
C6—C5—H5	119.6	C19—C18—H18	120.0
C10—C5—H5	119.6	C17—C18—H18	120.0
C5—C6—C7	120.9 (3)	C20—C19—C18	120.2 (3)
C5—C6—H6	119.5	C20—C19—H19	119.9
C7—C6—H6	119.5	C18—C19—H19	119.9
C8—C7—C6	120.0 (3)	C19—C20—C21	120.1 (2)
C8—C7—H7	120.0	C19—C20—H20	119.9
C6—C7—H7	120.0	C21—C20—H20	119.9
C7—C8—C9	120.0 (2)	C20—C21—C22	120.6 (3)
C7—C8—P1	125.31 (19)	C20—C21—H21	119.7
C9—C8—P1	114.59 (18)	C22—C21—H21	119.7
N1—C9—C8	119.1 (2)	C21—C22—C17	119.3 (2)
N1—C9—C10	121.2 (2)	C21—C22—H22	120.4
C8—C9—C10	119.7 (2)	C17—C22—H22	120.4
			
C9—N1—C2—C3	3.4 (4)	C3—C4—C10—C9	2.3 (4)
Pd1—N1—C2—C3	−175.5 (2)	C17—P1—C11—C12	28.7 (2)
N1—C2—C3—C4	0.5 (4)	C8—P1—C11—C12	143.3 (2)
C2—C3—C4—C10	−3.3 (4)	Pd1—P1—C11—C12	−103.4 (2)
C10—C5—C6—C7	0.9 (4)	C17—P1—C11—C16	−156.7 (2)
C5—C6—C7—C8	−0.2 (4)	C8—P1—C11—C16	−42.1 (2)
C6—C7—C8—C9	−0.6 (4)	Pd1—P1—C11—C16	71.2 (2)
C6—C7—C8—P1	−176.4 (2)	C16—C11—C12—C13	0.6 (4)
C11—P1—C8—C7	−50.7 (2)	P1—C11—C12—C13	175.2 (2)
C17—P1—C8—C7	64.7 (2)	C11—C12—C13—C14	−1.0 (4)
Pd1—P1—C8—C7	−175.1 (2)	C12—C13—C14—C15	0.6 (4)
C11—P1—C8—C9	133.35 (18)	C13—C14—C15—C16	0.1 (4)
C17—P1—C8—C9	−111.23 (18)	C14—C15—C16—C11	−0.5 (4)
Pd1—P1—C8—C9	8.88 (18)	C12—C11—C16—C15	0.1 (4)
C2—N1—C9—C8	175.3 (2)	P1—C11—C16—C15	−174.6 (2)
Pd1—N1—C9—C8	−5.7 (3)	C11—P1—C17—C22	52.8 (2)
C2—N1—C9—C10	−4.5 (3)	C8—P1—C17—C22	−61.3 (2)
Pd1—N1—C9—C10	174.54 (17)	Pd1—P1—C17—C22	−172.94 (17)
C7—C8—C9—N1	−179.2 (2)	C11—P1—C17—C18	−133.20 (19)
P1—C8—C9—N1	−3.0 (3)	C8—P1—C17—C18	112.7 (2)
C7—C8—C9—C10	0.6 (3)	Pd1—P1—C17—C18	1.0 (2)
P1—C8—C9—C10	176.80 (18)	C22—C17—C18—C19	1.0 (4)
C6—C5—C10—C9	−0.9 (4)	P1—C17—C18—C19	−173.1 (2)
C6—C5—C10—C4	177.3 (3)	C17—C18—C19—C20	−0.6 (4)
N1—C9—C10—C5	179.9 (2)	C18—C19—C20—C21	−0.4 (4)
C8—C9—C10—C5	0.1 (3)	C19—C20—C21—C22	1.2 (4)
N1—C9—C10—C4	1.7 (3)	C20—C21—C22—C17	−0.8 (4)
C8—C9—C10—C4	−178.1 (2)	C18—C17—C22—C21	−0.2 (4)
C3—C4—C10—C5	−175.9 (3)	P1—C17—C22—C21	173.7 (2)

### (2) Dichlorido(8-diphenylphosphanylquinoline)platinum(II) dichloromethane monosolvate Crystal data

**Table:**

[PtCl_2_(C_21_H_16_NP)]·CH_2_Cl_2_	*F*(000) = 1272
*M**_r_* = 664.23	*D*_x_ = 1.966 Mg m^−^^3^
Monoclinic, *P*2_1_/*c*	Mo *K*α radiation, λ = 0.71075 Å
*a* = 13.9280 (5) Å	Cell parameters from 12710 reflections
*b* = 9.2371 (3) Å	θ = 3.0–27.4°
*c* = 17.8941 (6) Å	µ = 6.81 mm^−^^1^
β = 102.8447 (10)°	*T* = 200 K
*V* = 2244.53 (13) Å^3^	Platelet, colorless
*Z* = 4	0.25 × 0.24 × 0.05 mm

### (2) Dichlorido(8-diphenylphosphanylquinoline)platinum(II) dichloromethane monosolvate Data collection

**Table:**

Rigaku R-AXIS RAPID diffractometer	5081 independent reflections
Radiation source: fine-focus sealed tube	4665 reflections with *I* > 2σ(*I*)
Detector resolution: 10.00 pixels mm^-1^	*R*_int_ = 0.028
ω scans	θ_max_ = 27.4°, θ_min_ = 3.0°
Absorption correction: numerical (*NUMABS*; Rigaku, 1999)	*h* = −18→18
*T*_min_ = 0.281, *T*_max_ = 0.727	*k* = −11→10
20995 measured reflections	*l* = −23→23

### (2) Dichlorido(8-diphenylphosphanylquinoline)platinum(II) dichloromethane monosolvate Refinement

**Table:**

Refinement on *F*^2^	Primary atom site location: structure-invariant direct methods
Least-squares matrix: full	Secondary atom site location: difference Fourier map
*R*[*F*^2^ > 2σ(*F*^2^)] = 0.017	Hydrogen site location: inferred from neighbouring sites
*wR*(*F*^2^) = 0.037	H-atom parameters constrained
*S* = 0.98	*w* = 1/[σ^2^(*F*_o_^2^) + (0.0073*P*)^2^ + 3.0468*P*] where *P* = (*F*_o_^2^ + 2*F*_c_^2^)/3
5081 reflections	(Δ/σ)_max_ = 0.003
262 parameters	Δρ_max_ = 0.58 e Å^−^^3^
0 restraints	Δρ_min_ = −0.60 e Å^−^^3^

### (2) Dichlorido(8-diphenylphosphanylquinoline)platinum(II) dichloromethane monosolvate Special details

**Table:**

Geometry. All e.s.d.'s (except the e.s.d. in the dihedral angle between two l.s. planes) are estimated using the full covariance matrix. The cell e.s.d.'s are taken into account individually in the estimation of e.s.d.'s in distances, angles and torsion angles; correlations between e.s.d.'s in cell parameters are only used when they are defined by crystal symmetry. An approximate (isotropic) treatment of cell e.s.d.'s is used for estimating e.s.d.'s involving l.s. planes.

### (2) Dichlorido(8-diphenylphosphanylquinoline)platinum(II) dichloromethane monosolvate Fractional atomic coordinates and isotropic or equivalent isotropic displacement parameters (Å^2^)

**Table:**

	*x*	*y*	*z*	*U*_iso_*/*U*_eq_	
Pt1	0.34435 (2)	0.08493 (2)	0.69614 (2)	0.02160 (3)	
Cl1	0.40053 (5)	−0.06049 (7)	0.80665 (3)	0.03408 (14)	
Cl2	0.20025 (5)	0.12939 (8)	0.73477 (4)	0.03670 (15)	
Cl3	0.19749 (8)	0.61854 (9)	0.43864 (4)	0.0575 (2)	
Cl4	0.04531 (7)	0.67431 (12)	0.30251 (5)	0.0619 (2)	
P1	0.29474 (5)	0.22285 (6)	0.59493 (3)	0.02219 (12)	
N1	0.46929 (15)	0.0549 (2)	0.65523 (11)	0.0237 (4)	
C1	0.1712 (2)	0.6794 (3)	0.34355 (16)	0.0393 (6)	
H1A	0.1953	0.7800	0.3420	0.047*	
H1B	0.2065	0.6182	0.3131	0.047*	
C2	0.5401 (2)	−0.0380 (3)	0.68572 (14)	0.0313 (6)	
H2	0.5313	−0.0960	0.7275	0.038*	
C3	0.6271 (2)	−0.0547 (3)	0.65944 (16)	0.0367 (6)	
H3	0.6758	−0.1220	0.6834	0.044*	
C4	0.6411 (2)	0.0261 (3)	0.59951 (15)	0.0342 (6)	
H4	0.7001	0.0164	0.5816	0.041*	
C5	0.5755 (2)	0.2104 (3)	0.49983 (15)	0.0352 (6)	
H5	0.6324	0.2022	0.4791	0.042*	
C6	0.5028 (2)	0.3044 (3)	0.46766 (15)	0.0374 (6)	
H6	0.5101	0.3626	0.4255	0.045*	
C7	0.4172 (2)	0.3161 (3)	0.49634 (14)	0.0319 (6)	
H7	0.3670	0.3821	0.4733	0.038*	
C8	0.40500 (18)	0.2329 (3)	0.55748 (13)	0.0241 (5)	
C9	0.48110 (18)	0.1374 (3)	0.59276 (13)	0.0239 (5)	
C10	0.5677 (2)	0.1246 (3)	0.56378 (14)	0.0283 (5)	
C11	0.2562 (2)	0.4050 (3)	0.61167 (13)	0.0274 (5)	
C12	0.1573 (2)	0.4345 (3)	0.60854 (17)	0.0403 (7)	
H12	0.1089	0.3618	0.5922	0.048*	
C13	0.1296 (3)	0.5704 (3)	0.6294 (2)	0.0524 (9)	
H13	0.0622	0.5909	0.6272	0.063*	
C14	0.2001 (3)	0.6758 (3)	0.65334 (17)	0.0481 (8)	
H14	0.1808	0.7685	0.6678	0.058*	
C15	0.2971 (3)	0.6479 (3)	0.65643 (16)	0.0440 (8)	
H15	0.3449	0.7213	0.6728	0.053*	
C16	0.3265 (2)	0.5121 (3)	0.63574 (13)	0.0318 (6)	
H16	0.3941	0.4929	0.6381	0.038*	
C17	0.20016 (18)	0.1489 (3)	0.51834 (13)	0.0249 (5)	
C18	0.1620 (2)	0.0112 (3)	0.52486 (15)	0.0305 (6)	
H18	0.1829	−0.0425	0.5709	0.037*	
C19	0.0931 (2)	−0.0475 (3)	0.46383 (17)	0.0386 (6)	
H19	0.0670	−0.1413	0.4685	0.046*	
C20	0.0623 (2)	0.0296 (3)	0.39660 (16)	0.0420 (7)	
H20	0.0150	−0.0107	0.3553	0.050*	
C21	0.1009 (2)	0.1660 (4)	0.38972 (16)	0.0450 (7)	
H21	0.0807	0.2186	0.3433	0.054*	
C22	0.1687 (2)	0.2259 (3)	0.45004 (15)	0.0383 (6)	
H22	0.1941	0.3200	0.4451	0.046*	

### (2) Dichlorido(8-diphenylphosphanylquinoline)platinum(II) dichloromethane monosolvate Atomic displacement parameters (Å^2^)

**Table:**

	*U*^11^	*U*^22^	*U*^33^	*U*^12^	*U*^13^	*U*^23^
Pt1	0.02203 (5)	0.02250 (5)	0.02056 (5)	−0.00063 (4)	0.00538 (3)	−0.00020 (3)
Cl1	0.0310 (4)	0.0435 (4)	0.0267 (3)	0.0012 (3)	0.0042 (2)	0.0108 (3)
Cl2	0.0304 (4)	0.0427 (4)	0.0423 (3)	0.0051 (3)	0.0193 (3)	0.0086 (3)
Cl3	0.0775 (7)	0.0463 (4)	0.0421 (4)	0.0134 (4)	−0.0009 (4)	0.0046 (3)
Cl4	0.0372 (5)	0.0920 (7)	0.0503 (4)	0.0008 (4)	−0.0034 (4)	−0.0148 (4)
P1	0.0220 (3)	0.0218 (3)	0.0232 (3)	0.0003 (2)	0.0059 (2)	0.0005 (2)
N1	0.0226 (11)	0.0256 (10)	0.0228 (9)	0.0005 (8)	0.0051 (8)	−0.0043 (8)
C1	0.0342 (17)	0.0445 (16)	0.0389 (14)	−0.0001 (13)	0.0073 (12)	−0.0018 (13)
C2	0.0304 (15)	0.0352 (14)	0.0272 (12)	0.0058 (11)	0.0044 (11)	−0.0013 (11)
C3	0.0298 (15)	0.0410 (15)	0.0380 (14)	0.0096 (12)	0.0046 (12)	−0.0075 (12)
C4	0.0260 (15)	0.0414 (15)	0.0365 (14)	−0.0005 (12)	0.0094 (11)	−0.0142 (12)
C5	0.0317 (16)	0.0429 (16)	0.0355 (13)	−0.0102 (12)	0.0173 (12)	−0.0097 (12)
C6	0.0428 (18)	0.0401 (15)	0.0333 (13)	−0.0084 (13)	0.0171 (12)	0.0016 (12)
C7	0.0351 (16)	0.0312 (13)	0.0299 (12)	−0.0027 (11)	0.0080 (11)	0.0010 (11)
C8	0.0228 (13)	0.0256 (12)	0.0245 (11)	−0.0032 (10)	0.0063 (9)	−0.0048 (9)
C9	0.0257 (13)	0.0250 (11)	0.0212 (11)	−0.0051 (10)	0.0057 (9)	−0.0074 (9)
C10	0.0255 (14)	0.0306 (13)	0.0297 (12)	−0.0058 (10)	0.0078 (10)	−0.0104 (10)
C11	0.0363 (15)	0.0233 (12)	0.0242 (11)	0.0019 (11)	0.0104 (10)	0.0017 (9)
C12	0.0383 (18)	0.0309 (14)	0.0558 (18)	0.0018 (12)	0.0191 (14)	−0.0023 (13)
C13	0.060 (2)	0.0383 (17)	0.068 (2)	0.0172 (16)	0.0349 (19)	0.0037 (15)
C14	0.085 (3)	0.0241 (14)	0.0436 (16)	0.0096 (15)	0.0329 (17)	0.0021 (12)
C15	0.076 (3)	0.0254 (13)	0.0333 (14)	−0.0086 (15)	0.0173 (15)	−0.0041 (11)
C16	0.0416 (17)	0.0291 (13)	0.0251 (12)	−0.0041 (12)	0.0086 (11)	0.0010 (10)
C17	0.0236 (13)	0.0237 (11)	0.0273 (11)	0.0027 (10)	0.0052 (10)	−0.0020 (9)
C18	0.0283 (15)	0.0304 (13)	0.0329 (13)	−0.0022 (11)	0.0072 (11)	−0.0004 (11)
C19	0.0331 (16)	0.0346 (14)	0.0483 (16)	−0.0072 (12)	0.0091 (13)	−0.0118 (13)
C20	0.0305 (16)	0.0529 (18)	0.0391 (15)	−0.0009 (14)	0.0002 (12)	−0.0158 (14)
C21	0.0432 (19)	0.0534 (19)	0.0322 (14)	0.0019 (15)	−0.0052 (13)	0.0027 (13)
C22	0.0399 (17)	0.0322 (14)	0.0375 (14)	−0.0011 (12)	−0.0026 (12)	0.0057 (12)

### (2) Dichlorido(8-diphenylphosphanylquinoline)platinum(II) dichloromethane monosolvate Geometric parameters (Å, º)

**Table:**

Pt1—N1	2.051 (2)	C7—H7	0.9500
Pt1—P1	2.1963 (6)	C8—C9	1.416 (3)
Pt1—Cl2	2.3002 (7)	C9—C10	1.420 (3)
Pt1—Cl1	2.3747 (6)	C11—C16	1.392 (4)
Cl3—C1	1.752 (3)	C11—C12	1.393 (4)
Cl4—C1	1.744 (3)	C12—C13	1.388 (4)
P1—C17	1.810 (3)	C12—H12	0.9500
P1—C8	1.809 (2)	C13—C14	1.381 (5)
P1—C11	1.811 (2)	C13—H13	0.9500
N1—C2	1.329 (3)	C14—C15	1.366 (5)
N1—C9	1.392 (3)	C14—H14	0.9500
C1—H1A	0.9900	C15—C16	1.394 (4)
C1—H1B	0.9900	C15—H15	0.9500
C2—C3	1.402 (4)	C16—H16	0.9500
C2—H2	0.9500	C17—C18	1.393 (4)
C3—C4	1.356 (4)	C17—C22	1.398 (3)
C3—H3	0.9500	C18—C19	1.393 (4)
C4—C10	1.411 (4)	C18—H18	0.9500
C4—H4	0.9500	C19—C20	1.381 (4)
C5—C6	1.361 (4)	C19—H19	0.9500
C5—C10	1.416 (4)	C20—C21	1.386 (4)
C5—H5	0.9500	C20—H20	0.9500
C6—C7	1.404 (4)	C21—C22	1.382 (4)
C6—H6	0.9500	C21—H21	0.9500
C7—C8	1.378 (3)	C22—H22	0.9500
			
N1—Pt1—P1	85.44 (6)	N1—C9—C8	119.3 (2)
N1—Pt1—Cl2	175.93 (6)	N1—C9—C10	120.6 (2)
P1—Pt1—Cl2	90.50 (2)	C8—C9—C10	120.1 (2)
N1—Pt1—Cl1	94.13 (6)	C4—C10—C9	118.5 (2)
P1—Pt1—Cl1	178.80 (2)	C4—C10—C5	123.3 (2)
Cl2—Pt1—Cl1	89.93 (2)	C9—C10—C5	118.2 (2)
C17—P1—C8	105.88 (11)	C16—C11—C12	119.6 (2)
C17—P1—C11	106.45 (12)	C16—C11—P1	119.9 (2)
C8—P1—C11	108.71 (12)	C12—C11—P1	120.1 (2)
C17—P1—Pt1	116.61 (8)	C13—C12—C11	119.8 (3)
C8—P1—Pt1	101.42 (8)	C13—C12—H12	120.1
C11—P1—Pt1	116.93 (8)	C11—C12—H12	120.1
C2—N1—C9	118.2 (2)	C14—C13—C12	120.1 (3)
C2—N1—Pt1	123.48 (17)	C14—C13—H13	120.0
C9—N1—Pt1	118.27 (16)	C12—C13—H13	120.0
Cl4—C1—Cl3	111.96 (17)	C15—C14—C13	120.5 (3)
Cl4—C1—H1A	109.2	C15—C14—H14	119.7
Cl3—C1—H1A	109.2	C13—C14—H14	119.7
Cl4—C1—H1B	109.2	C14—C15—C16	120.3 (3)
Cl3—C1—H1B	109.2	C14—C15—H15	119.8
H1A—C1—H1B	107.9	C16—C15—H15	119.8
N1—C2—C3	123.4 (2)	C11—C16—C15	119.6 (3)
N1—C2—H2	118.3	C11—C16—H16	120.2
C3—C2—H2	118.3	C15—C16—H16	120.2
C4—C3—C2	119.5 (3)	C18—C17—C22	119.1 (2)
C4—C3—H3	120.3	C18—C17—P1	120.54 (19)
C2—C3—H3	120.3	C22—C17—P1	120.2 (2)
C3—C4—C10	119.7 (3)	C17—C18—C19	119.9 (2)
C3—C4—H4	120.1	C17—C18—H18	120.0
C10—C4—H4	120.1	C19—C18—H18	120.0
C6—C5—C10	121.0 (3)	C20—C19—C18	120.5 (3)
C6—C5—H5	119.5	C20—C19—H19	119.7
C10—C5—H5	119.5	C18—C19—H19	119.7
C5—C6—C7	120.5 (3)	C19—C20—C21	119.6 (3)
C5—C6—H6	119.8	C19—C20—H20	120.2
C7—C6—H6	119.8	C21—C20—H20	120.2
C8—C7—C6	120.8 (3)	C22—C21—C20	120.4 (3)
C8—C7—H7	119.6	C22—C21—H21	119.8
C6—C7—H7	119.6	C20—C21—H21	119.8
C7—C8—C9	119.3 (2)	C21—C22—C17	120.4 (3)
C7—C8—P1	126.0 (2)	C21—C22—H22	119.8
C9—C8—P1	114.57 (17)	C17—C22—H22	119.8
			
C9—N1—C2—C3	1.6 (4)	C6—C5—C10—C9	1.0 (4)
Pt1—N1—C2—C3	−177.6 (2)	C17—P1—C11—C16	−147.98 (19)
N1—C2—C3—C4	−0.5 (4)	C8—P1—C11—C16	−34.3 (2)
C2—C3—C4—C10	−0.7 (4)	Pt1—P1—C11—C16	79.7 (2)
C10—C5—C6—C7	−1.4 (4)	C17—P1—C11—C12	38.9 (2)
C5—C6—C7—C8	0.0 (4)	C8—P1—C11—C12	152.6 (2)
C6—C7—C8—C9	1.6 (4)	Pt1—P1—C11—C12	−93.4 (2)
C6—C7—C8—P1	−173.7 (2)	C16—C11—C12—C13	0.0 (4)
C17—P1—C8—C7	62.9 (2)	P1—C11—C12—C13	173.1 (2)
C11—P1—C8—C7	−51.1 (2)	C11—C12—C13—C14	−0.1 (5)
Pt1—P1—C8—C7	−174.9 (2)	C12—C13—C14—C15	0.3 (5)
C17—P1—C8—C9	−112.53 (18)	C13—C14—C15—C16	−0.3 (4)
C11—P1—C8—C9	133.44 (18)	C12—C11—C16—C15	0.0 (4)
Pt1—P1—C8—C9	9.65 (18)	P1—C11—C16—C15	−173.17 (19)
C2—N1—C9—C8	177.6 (2)	C14—C15—C16—C11	0.2 (4)
Pt1—N1—C9—C8	−3.1 (3)	C8—P1—C17—C18	112.0 (2)
C2—N1—C9—C10	−1.5 (3)	C11—P1—C17—C18	−132.4 (2)
Pt1—N1—C9—C10	177.82 (17)	Pt1—P1—C17—C18	0.1 (2)
C7—C8—C9—N1	179.1 (2)	C8—P1—C17—C22	−64.2 (2)
P1—C8—C9—N1	−5.1 (3)	C11—P1—C17—C22	51.3 (2)
C7—C8—C9—C10	−1.9 (3)	Pt1—P1—C17—C22	−176.13 (19)
P1—C8—C9—C10	173.94 (17)	C22—C17—C18—C19	−0.3 (4)
C3—C4—C10—C9	0.8 (4)	P1—C17—C18—C19	−176.5 (2)
C3—C4—C10—C5	−178.5 (2)	C17—C18—C19—C20	0.2 (4)
N1—C9—C10—C4	0.3 (3)	C18—C19—C20—C21	0.4 (5)
C8—C9—C10—C4	−178.7 (2)	C19—C20—C21—C22	−1.0 (5)
N1—C9—C10—C5	179.6 (2)	C20—C21—C22—C17	0.9 (5)
C8—C9—C10—C5	0.6 (3)	C18—C17—C22—C21	−0.2 (4)
C6—C5—C10—C4	−179.7 (3)	P1—C17—C22—C21	176.0 (2)

### (3) cis-Dichloridobis(8-diphenylphosphanylquinoline)rhodium(III) hexafluoridophosphate dichloromethane/methanol hemisolvate Crystal data

**Table:**

[RhCl_2_(C_21_H_16_NP)_2_](PF_6_)·0.5CH_2_Cl_2_·0.5CH_4_O	*Z* = 2
*M**_r_* = 1003.90	*F*(000) = 1012
Triclinic, *P*1	*D*_x_ = 1.621 Mg m^−^^3^
*a* = 9.841 (5) Å	Mo *K*α radiation, λ = 0.71075 Å
*b* = 13.825 (6) Å	Cell parameters from 15480 reflections
*c* = 16.167 (8) Å	θ = 3.1–27.5°
α = 87.307 (19)°	µ = 0.79 mm^−^^1^
β = 81.80 (2)°	*T* = 200 K
γ = 70.819 (18)°	Prism, colorless
*V* = 2056.2 (17) Å^3^	0.20 × 0.20 × 0.15 mm

### (3) cis-Dichloridobis(8-diphenylphosphanylquinoline)rhodium(III) hexafluoridophosphate dichloromethane/methanol hemisolvate Data collection

**Table:**

Rigaku R-AXIS RAPID diffractometer	9338 independent reflections
Radiation source: fine-focus sealed tube	7245 reflections with *I* > 2σ(*I*)
Detector resolution: 10.00 pixels mm^-1^	*R*_int_ = 0.046
ω scans	θ_max_ = 27.5°, θ_min_ = 3.1°
Absorption correction: multi-scan (*ABSCOR.*; Rigaku, 1995)	*h* = −12→12
*T*_min_ = 0.848, *T*_max_ = 0.882	*k* = −17→17
20385 measured reflections	*l* = −20→20

### (3) cis-Dichloridobis(8-diphenylphosphanylquinoline)rhodium(III) hexafluoridophosphate dichloromethane/methanol hemisolvate Refinement

**Table:**

Refinement on *F*^2^	Primary atom site location: heavy-atom method
Least-squares matrix: full	Secondary atom site location: difference Fourier map
*R*[*F*^2^ > 2σ(*F*^2^)] = 0.044	Hydrogen site location: inferred from neighbouring sites
*wR*(*F*^2^) = 0.120	H-atom parameters constrained
*S* = 1.06	*w* = 1/[σ^2^(*F*_o_^2^) + (0.064*P*)^2^] where *P* = (*F*_o_^2^ + 2*F*_c_^2^)/3
9338 reflections	(Δ/σ)_max_ = 0.002
605 parameters	Δρ_max_ = 0.68 e Å^−^^3^
20 restraints	Δρ_min_ = −1.03 e Å^−^^3^

### (3) cis-Dichloridobis(8-diphenylphosphanylquinoline)rhodium(III) hexafluoridophosphate dichloromethane/methanol hemisolvate Special details

**Table:**

Experimental. The ^31^P NMR spectrum of 3 in CD_3_CN (400 MHz, 22 °C) showed two doublet of doublets resonances at δ 38.66 (J_Rh–P_ = 110, J_P–P_ = 21 Hz) and 40.47 (J_Rh–P_ = 113 Hz).
Geometry. All e.s.d.'s (except the e.s.d. in the dihedral angle between two l.s. planes) are estimated using the full covariance matrix. The cell e.s.d.'s are taken into account individually in the estimation of e.s.d.'s in distances, angles and torsion angles; correlations between e.s.d.'s in cell parameters are only used when they are defined by crystal symmetry. An approximate (isotropic) treatment of cell e.s.d.'s is used for estimating e.s.d.'s involving l.s. planes.

### (3) cis-Dichloridobis(8-diphenylphosphanylquinoline)rhodium(III) hexafluoridophosphate dichloromethane/methanol hemisolvate Fractional atomic coordinates and isotropic or equivalent isotropic displacement parameters (Å^2^)

**Table:**

	*x*	*y*	*z*	*U*_iso_*/*U*_eq_	Occ. (<1)
Rh1	0.81791 (2)	0.88958 (2)	0.68292 (2)	0.02967 (9)	
Cl1	0.92494 (8)	0.90055 (6)	0.54278 (4)	0.03774 (17)	
Cl2	1.04776 (8)	0.82096 (6)	0.72496 (4)	0.03852 (17)	
P1	0.71063 (8)	0.90351 (6)	0.81912 (4)	0.03163 (17)	
P2	0.80756 (8)	0.73608 (6)	0.64925 (4)	0.03480 (18)	
P3	0.55681 (15)	0.62928 (12)	0.29883 (9)	0.0909 (4)	
F1A	0.6674 (12)	0.5251 (7)	0.3142 (9)	0.163 (6)	0.613 (14)
F2A	0.4337 (9)	0.5838 (8)	0.3072 (9)	0.132 (4)	0.613 (14)
F3A	0.601 (2)	0.6117 (9)	0.2058 (4)	0.182 (6)	0.613 (14)
F4A	0.559 (3)	0.633 (2)	0.3936 (6)	0.322 (12)	0.613 (14)
F5A	0.6781 (9)	0.6811 (7)	0.2764 (13)	0.174 (5)	0.613 (14)
F6A	0.4534 (11)	0.7384 (6)	0.2966 (15)	0.211 (8)	0.613 (14)
F1B	0.530 (4)	0.5418 (19)	0.356 (2)	0.300 (16)	0.387 (14)
F2B	0.451 (2)	0.6177 (18)	0.240 (2)	0.208 (10)	0.387 (14)
F3B	0.6695 (18)	0.5392 (19)	0.249 (2)	0.212 (13)	0.387 (14)
F4B	0.449 (2)	0.6981 (12)	0.3668 (10)	0.147 (7)	0.387 (14)
F5B	0.6769 (14)	0.6458 (17)	0.3408 (15)	0.163 (9)	0.387 (14)
F6B	0.522 (3)	0.7243 (11)	0.2443 (9)	0.232 (15)	0.387 (14)
N1	0.8319 (3)	1.03784 (18)	0.71011 (14)	0.0333 (5)	
N11	0.6185 (3)	0.9470 (2)	0.64031 (13)	0.0382 (6)	
Cl3A	0.3228 (12)	0.6504 (8)	0.8186 (7)	0.322 (5)	0.5
Cl4A	0.3702 (13)	0.5700 (9)	0.9744 (6)	0.407 (8)	0.5
C1A	0.289 (2)	0.5586 (14)	0.8897 (10)	0.307 (11)	0.5
H1A	0.1829	0.5731	0.9058	0.369*	0.5
H1B	0.3314	0.4890	0.8655	0.369*	0.5
O1B	0.3204 (14)	0.7010 (12)	0.8793 (10)	0.213 (8)	0.5
H1F	0.3593	0.7057	0.8302	0.256*	0.5
C1B	0.326 (2)	0.5987 (17)	0.8956 (10)	0.227 (11)	0.5
H1C	0.2739	0.5942	0.9514	0.341*	0.5
H1E	0.4273	0.5547	0.8932	0.341*	0.5
H1D	0.2801	0.5761	0.8537	0.341*	0.5
C2	0.8789 (4)	1.0945 (2)	0.6523 (2)	0.0478 (8)	
H2	0.8939	1.0739	0.5956	0.057*	
C3	0.9078 (4)	1.1828 (3)	0.6702 (2)	0.0567 (9)	
H3	0.9431	1.2203	0.6264	0.068*	
C4	0.8852 (4)	1.2148 (3)	0.7497 (2)	0.0571 (10)	
H4	0.9020	1.2760	0.7624	0.069*	
C5	0.8172 (5)	1.1806 (3)	0.8995 (2)	0.0696 (12)	
H5	0.8330	1.2406	0.9159	0.083*	
C6	0.7766 (5)	1.1198 (4)	0.9585 (2)	0.0743 (13)	
H6	0.7667	1.1368	1.0157	0.089*	
C7	0.7488 (4)	1.0322 (3)	0.93688 (19)	0.0542 (9)	
H7	0.7205	0.9901	0.9794	0.065*	
C8	0.7622 (3)	1.0064 (2)	0.85439 (17)	0.0377 (7)	
C9	0.8102 (3)	1.0674 (2)	0.79179 (18)	0.0369 (6)	
C10	0.8366 (4)	1.1571 (3)	0.8139 (2)	0.0484 (8)	
C12	0.5505 (4)	1.0465 (3)	0.63529 (19)	0.0525 (9)	
H12	0.5979	1.0922	0.6490	0.063*	
C13	0.4129 (4)	1.0875 (4)	0.6109 (2)	0.0713 (13)	
H13	0.3684	1.1596	0.6081	0.086*	
C14	0.3440 (4)	1.0250 (4)	0.5916 (2)	0.0761 (15)	
H14	0.2496	1.0525	0.5755	0.091*	
C15	0.3453 (5)	0.8472 (6)	0.5772 (3)	0.0870 (18)	
H15	0.2503	0.8714	0.5618	0.104*	
C16	0.4156 (6)	0.7439 (6)	0.5816 (3)	0.098 (2)	
H16	0.3687	0.6971	0.5697	0.118*	
C17	0.5564 (4)	0.7070 (4)	0.6036 (3)	0.0723 (12)	
H17	0.6048	0.6353	0.6055	0.087*	
C18	0.6254 (4)	0.7737 (3)	0.62245 (19)	0.0495 (9)	
C19	0.5529 (3)	0.8803 (3)	0.61891 (17)	0.0467 (8)	
C20	0.4116 (4)	0.9172 (4)	0.59511 (19)	0.0615 (11)	
C21	0.5141 (3)	0.9536 (3)	0.82721 (17)	0.0432 (8)	
C22	0.4385 (4)	1.0554 (3)	0.8417 (2)	0.0644 (11)	
H22	0.4881	1.1015	0.8512	0.077*	
C23	0.2882 (6)	1.0909 (5)	0.8423 (3)	0.107 (2)	
H23	0.2347	1.1613	0.8522	0.129*	
C24	0.2165 (6)	1.0214 (8)	0.8282 (3)	0.125 (3)	
H24	0.1141	1.0447	0.8291	0.151*	
C25	0.2927 (6)	0.9224 (7)	0.8134 (3)	0.104 (2)	
H25	0.2442	0.8760	0.8030	0.125*	
C26	0.4391 (4)	0.8879 (4)	0.8133 (2)	0.0639 (11)	
H26	0.4911	0.8174	0.8035	0.077*	
C27	0.7478 (4)	0.8059 (2)	0.89991 (17)	0.0428 (7)	
C28	0.6348 (5)	0.7924 (3)	0.9570 (2)	0.0645 (11)	
H28	0.5373	0.8325	0.9520	0.077*	
C29	0.6633 (6)	0.7216 (4)	1.0204 (3)	0.0891 (16)	
H29	0.5859	0.7139	1.0596	0.107*	
C30	0.8036 (7)	0.6625 (4)	1.0267 (3)	0.1008 (19)	
H30	0.8233	0.6127	1.0699	0.121*	
C31	0.9143 (6)	0.6745 (4)	0.9719 (3)	0.102 (2)	
H31	1.0112	0.6331	0.9769	0.122*	
C32	0.8874 (5)	0.7475 (4)	0.9078 (2)	0.0761 (14)	
H32	0.9659	0.7562	0.8701	0.091*	
C33	0.9307 (3)	0.6735 (2)	0.55799 (18)	0.0373 (7)	
C34	1.0796 (3)	0.6486 (2)	0.55612 (19)	0.0437 (7)	
H34	1.1176	0.6639	0.6028	0.052*	
C35	1.1731 (4)	0.6015 (3)	0.4863 (2)	0.0505 (8)	
H35	1.2750	0.5846	0.4852	0.061*	
C36	1.1187 (4)	0.5794 (2)	0.4188 (2)	0.0487 (8)	
H36	1.1828	0.5464	0.3713	0.058*	
C37	0.9725 (4)	0.6047 (2)	0.41995 (19)	0.0481 (8)	
H37	0.9356	0.5894	0.3728	0.058*	
C38	0.8767 (4)	0.6523 (2)	0.48863 (18)	0.0427 (7)	
H38	0.7750	0.6703	0.4883	0.051*	
C39	0.8269 (4)	0.6356 (2)	0.72758 (18)	0.0407 (7)	
C40	0.7092 (5)	0.6125 (3)	0.7699 (3)	0.0689 (12)	
H40	0.6136	0.6507	0.7595	0.083*	
C41	0.7312 (6)	0.5334 (3)	0.8277 (3)	0.0844 (15)	
H41	0.6498	0.5179	0.8565	0.101*	
C42	0.8658 (6)	0.4779 (3)	0.8439 (2)	0.0714 (12)	
H42	0.8781	0.4249	0.8845	0.086*	
C43	0.9853 (6)	0.4982 (3)	0.8016 (2)	0.0724 (12)	
H43	1.0802	0.4584	0.8121	0.087*	
C44	0.9657 (4)	0.5771 (3)	0.7437 (2)	0.0597 (10)	
H44	1.0479	0.5915	0.7147	0.072*	

### (3) cis-Dichloridobis(8-diphenylphosphanylquinoline)rhodium(III) hexafluoridophosphate dichloromethane/methanol hemisolvate Atomic displacement parameters (Å^2^)

**Table:**

	*U*^11^	*U*^22^	*U*^33^	*U*^12^	*U*^13^	*U*^23^
Rh1	0.03129 (14)	0.03778 (14)	0.02306 (12)	−0.01406 (10)	−0.00624 (8)	−0.00226 (9)
Cl1	0.0437 (4)	0.0465 (4)	0.0252 (3)	−0.0185 (3)	−0.0016 (3)	−0.0013 (3)
Cl2	0.0332 (4)	0.0482 (4)	0.0393 (4)	−0.0168 (3)	−0.0119 (3)	−0.0035 (3)
P1	0.0335 (4)	0.0417 (4)	0.0227 (3)	−0.0152 (3)	−0.0058 (3)	−0.0020 (3)
P2	0.0358 (4)	0.0452 (4)	0.0305 (4)	−0.0218 (4)	−0.0038 (3)	−0.0078 (3)
P3	0.0619 (8)	0.1049 (11)	0.0944 (10)	−0.0099 (8)	−0.0086 (7)	−0.0199 (9)
F1A	0.099 (7)	0.145 (8)	0.206 (11)	0.007 (6)	−0.030 (8)	0.076 (9)
F2A	0.072 (4)	0.155 (8)	0.176 (9)	−0.058 (5)	0.023 (5)	−0.025 (7)
F3A	0.281 (16)	0.160 (11)	0.067 (4)	−0.040 (10)	0.018 (6)	0.022 (5)
F4A	0.30 (2)	0.51 (4)	0.108 (8)	−0.04 (3)	−0.068 (11)	−0.103 (13)
F5A	0.106 (7)	0.117 (6)	0.290 (16)	−0.033 (5)	−0.008 (8)	0.011 (9)
F6A	0.088 (6)	0.112 (7)	0.42 (2)	0.032 (5)	−0.092 (10)	−0.112 (12)
F1B	0.28 (4)	0.20 (2)	0.42 (4)	−0.11 (2)	−0.03 (4)	0.16 (3)
F2B	0.164 (18)	0.21 (2)	0.26 (2)	−0.038 (13)	−0.078 (18)	−0.12 (2)
F3B	0.115 (14)	0.20 (2)	0.30 (3)	−0.040 (14)	0.08 (2)	−0.14 (2)
F4B	0.140 (13)	0.143 (12)	0.121 (11)	−0.015 (9)	0.044 (9)	−0.052 (8)
F5B	0.079 (9)	0.22 (2)	0.184 (17)	−0.009 (10)	−0.078 (11)	−0.073 (14)
F6B	0.43 (4)	0.145 (17)	0.110 (10)	−0.11 (2)	−0.001 (15)	0.079 (12)
N1	0.0396 (14)	0.0320 (12)	0.0291 (11)	−0.0126 (11)	−0.0045 (10)	−0.0033 (10)
N11	0.0332 (14)	0.0595 (16)	0.0202 (11)	−0.0122 (12)	−0.0057 (9)	0.0011 (11)
Cl3A	0.302 (11)	0.251 (10)	0.445 (14)	−0.111 (8)	−0.131 (12)	0.083 (10)
Cl4A	0.480 (19)	0.444 (17)	0.221 (8)	−0.130 (12)	0.146 (10)	0.022 (9)
C1A	0.35 (3)	0.218 (19)	0.33 (2)	−0.15 (2)	0.233 (17)	−0.174 (15)
O1B	0.118 (9)	0.287 (16)	0.257 (16)	−0.110 (10)	0.061 (10)	−0.168 (13)
C1B	0.26 (2)	0.48 (3)	0.114 (11)	−0.33 (2)	−0.086 (12)	0.119 (16)
C2	0.067 (2)	0.0415 (17)	0.0344 (15)	−0.0181 (16)	−0.0036 (15)	0.0000 (14)
C3	0.080 (3)	0.0396 (18)	0.052 (2)	−0.0268 (18)	0.0016 (18)	0.0005 (16)
C4	0.075 (3)	0.0420 (18)	0.060 (2)	−0.0295 (19)	−0.0005 (19)	−0.0092 (17)
C5	0.100 (3)	0.072 (3)	0.053 (2)	−0.054 (3)	0.008 (2)	−0.027 (2)
C6	0.111 (4)	0.093 (3)	0.0392 (19)	−0.064 (3)	0.006 (2)	−0.026 (2)
C7	0.070 (2)	0.071 (2)	0.0324 (15)	−0.040 (2)	0.0014 (15)	−0.0128 (16)
C8	0.0358 (16)	0.0489 (17)	0.0311 (14)	−0.0173 (14)	−0.0017 (12)	−0.0085 (13)
C9	0.0331 (16)	0.0410 (16)	0.0358 (15)	−0.0096 (13)	−0.0041 (12)	−0.0090 (13)
C10	0.056 (2)	0.0469 (19)	0.0454 (18)	−0.0220 (17)	0.0004 (15)	−0.0139 (15)
C12	0.047 (2)	0.067 (2)	0.0326 (16)	−0.0030 (17)	−0.0095 (14)	0.0074 (16)
C13	0.046 (2)	0.100 (3)	0.044 (2)	0.009 (2)	−0.0103 (17)	0.015 (2)
C14	0.034 (2)	0.142 (5)	0.0343 (18)	−0.003 (3)	−0.0126 (15)	0.010 (2)
C15	0.038 (2)	0.180 (6)	0.051 (2)	−0.044 (3)	−0.0051 (18)	−0.029 (3)
C16	0.064 (3)	0.184 (6)	0.077 (3)	−0.079 (4)	−0.001 (2)	−0.049 (4)
C17	0.054 (2)	0.106 (3)	0.075 (3)	−0.048 (2)	0.000 (2)	−0.036 (2)
C18	0.0394 (19)	0.082 (3)	0.0365 (16)	−0.0320 (18)	−0.0013 (13)	−0.0200 (16)
C19	0.0312 (17)	0.087 (3)	0.0233 (13)	−0.0201 (17)	−0.0037 (11)	−0.0091 (15)
C20	0.0347 (19)	0.123 (4)	0.0258 (15)	−0.023 (2)	−0.0073 (13)	−0.0020 (19)
C21	0.0369 (17)	0.071 (2)	0.0236 (13)	−0.0205 (16)	−0.0054 (12)	0.0023 (14)
C22	0.046 (2)	0.084 (3)	0.051 (2)	−0.005 (2)	−0.0067 (17)	0.006 (2)
C23	0.058 (3)	0.150 (6)	0.069 (3)	0.024 (3)	−0.003 (2)	0.012 (3)
C24	0.034 (3)	0.271 (10)	0.056 (3)	−0.029 (4)	−0.011 (2)	0.009 (4)
C25	0.050 (3)	0.241 (8)	0.044 (2)	−0.075 (4)	−0.003 (2)	−0.022 (3)
C26	0.051 (2)	0.120 (4)	0.0341 (16)	−0.047 (2)	−0.0011 (15)	−0.0148 (19)
C27	0.059 (2)	0.0453 (17)	0.0256 (13)	−0.0170 (16)	−0.0089 (13)	−0.0009 (13)
C28	0.075 (3)	0.074 (3)	0.0450 (19)	−0.028 (2)	−0.0082 (18)	0.0157 (19)
C29	0.124 (5)	0.091 (4)	0.048 (2)	−0.037 (3)	−0.001 (3)	0.025 (2)
C30	0.149 (5)	0.081 (3)	0.047 (2)	−0.008 (3)	−0.005 (3)	0.021 (2)
C31	0.099 (4)	0.110 (4)	0.051 (2)	0.026 (3)	−0.012 (3)	0.017 (3)
C32	0.068 (3)	0.101 (3)	0.0350 (18)	0.003 (2)	−0.0040 (18)	0.014 (2)
C33	0.0431 (18)	0.0379 (15)	0.0359 (15)	−0.0207 (14)	−0.0012 (13)	−0.0082 (13)
C34	0.0431 (19)	0.0511 (19)	0.0405 (16)	−0.0209 (15)	0.0000 (13)	−0.0132 (14)
C35	0.050 (2)	0.0480 (19)	0.054 (2)	−0.0198 (16)	0.0054 (16)	−0.0127 (16)
C36	0.068 (2)	0.0366 (16)	0.0392 (16)	−0.0194 (16)	0.0109 (16)	−0.0116 (14)
C37	0.072 (3)	0.0428 (17)	0.0344 (15)	−0.0260 (17)	−0.0039 (15)	−0.0079 (14)
C38	0.055 (2)	0.0463 (17)	0.0341 (15)	−0.0263 (16)	−0.0058 (14)	−0.0066 (13)
C39	0.054 (2)	0.0420 (17)	0.0341 (15)	−0.0283 (15)	0.0012 (13)	−0.0080 (13)
C40	0.066 (3)	0.052 (2)	0.083 (3)	−0.023 (2)	0.014 (2)	0.008 (2)
C41	0.093 (4)	0.070 (3)	0.087 (3)	−0.040 (3)	0.027 (3)	0.011 (3)
C42	0.118 (4)	0.055 (2)	0.049 (2)	−0.042 (3)	−0.004 (2)	0.0045 (19)
C43	0.099 (4)	0.081 (3)	0.050 (2)	−0.042 (3)	−0.025 (2)	0.017 (2)
C44	0.067 (3)	0.083 (3)	0.0443 (19)	−0.042 (2)	−0.0161 (17)	0.0071 (19)

### (3) cis-Dichloridobis(8-diphenylphosphanylquinoline)rhodium(III) hexafluoridophosphate dichloromethane/methanol hemisolvate Geometric parameters (Å, º)

**Table:**

Rh1—N11	2.065 (2)	C14—C20	1.420 (7)
Rh1—N1	2.168 (2)	C14—H14	0.9500
Rh1—P2	2.2531 (8)	C15—C16	1.370 (8)
Rh1—P1	2.2897 (7)	C15—C20	1.392 (7)
Rh1—Cl2	2.3338 (7)	C15—H15	0.9500
Rh1—Cl1	2.3787 (6)	C16—C17	1.402 (7)
P1—C8	1.800 (3)	C16—H16	0.9500
P1—C21	1.815 (3)	C17—C18	1.378 (5)
P1—C27	1.818 (3)	C17—H17	0.9500
P2—C18	1.806 (3)	C18—C19	1.413 (5)
P2—C39	1.816 (3)	C19—C20	1.418 (5)
P2—C33	1.821 (3)	C21—C22	1.372 (5)
P3—F3A	1.513 (6)	C21—C26	1.389 (5)
P3—F6B	1.518 (8)	C22—C23	1.396 (6)
P3—F6A	1.519 (7)	C22—H22	0.9500
P3—F2A	1.526 (6)	C23—C24	1.409 (10)
P3—F5B	1.526 (8)	C23—H23	0.9500
P3—F1A	1.528 (6)	C24—C25	1.341 (9)
P3—F4B	1.529 (8)	C24—H24	0.9500
P3—F3B	1.538 (8)	C25—C26	1.360 (6)
P3—F4A	1.539 (7)	C25—H25	0.9500
P3—F2B	1.552 (8)	C26—H26	0.9500
P3—F1B	1.558 (9)	C27—C32	1.367 (5)
P3—F5A	1.576 (7)	C27—C28	1.397 (5)
N1—C2	1.322 (4)	C28—C29	1.376 (5)
N1—C9	1.367 (4)	C28—H28	0.9500
N11—C12	1.321 (4)	C29—C30	1.370 (7)
N11—C19	1.368 (4)	C29—H29	0.9500
Cl3A—C1A	1.754 (10)	C30—C31	1.353 (7)
Cl4A—C1A	1.721 (10)	C30—H30	0.9500
C1A—H1A	0.9900	C31—C32	1.402 (5)
C1A—H1B	0.9900	C31—H31	0.9500
O1B—C1B	1.412 (16)	C32—H32	0.9500
O1B—H1F	0.8400	C33—C34	1.387 (4)
C1B—H1C	0.9800	C33—C38	1.388 (4)
C1B—H1E	0.9800	C34—C35	1.387 (4)
C1B—H1D	0.9800	C34—H34	0.9500
C2—C3	1.392 (5)	C35—C36	1.370 (5)
C2—H2	0.9500	C35—H35	0.9500
C3—C4	1.344 (5)	C36—C37	1.361 (5)
C3—H3	0.9500	C36—H36	0.9500
C4—C10	1.405 (5)	C37—C38	1.385 (4)
C4—H4	0.9500	C37—H37	0.9500
C5—C6	1.346 (5)	C38—H38	0.9500
C5—C10	1.407 (5)	C39—C40	1.381 (5)
C5—H5	0.9500	C39—C44	1.395 (5)
C6—C7	1.396 (5)	C40—C41	1.385 (6)
C6—H6	0.9500	C40—H40	0.9500
C7—C8	1.373 (4)	C41—C42	1.351 (6)
C7—H7	0.9500	C41—H41	0.9500
C8—C9	1.416 (4)	C42—C43	1.377 (6)
C9—C10	1.418 (4)	C42—H42	0.9500
C12—C13	1.392 (5)	C43—C44	1.387 (5)
C12—H12	0.9500	C43—H43	0.9500
C13—C14	1.332 (7)	C44—H44	0.9500
C13—H13	0.9500		
			
N11—Rh1—N1	95.37 (10)	C4—C10—C9	118.3 (3)
N11—Rh1—P2	84.53 (8)	C5—C10—C9	117.6 (3)
N1—Rh1—P2	177.65 (6)	N11—C12—C13	123.2 (4)
N11—Rh1—P1	91.56 (6)	N11—C12—H12	118.4
N1—Rh1—P1	81.81 (6)	C13—C12—H12	118.4
P2—Rh1—P1	100.55 (3)	C14—C13—C12	119.5 (4)
N11—Rh1—Cl2	177.29 (7)	C14—C13—H13	120.2
N1—Rh1—Cl2	86.06 (7)	C12—C13—H13	120.2
P2—Rh1—Cl2	93.94 (3)	C13—C14—C20	120.1 (4)
P1—Rh1—Cl2	90.92 (3)	C13—C14—H14	120.0
N11—Rh1—Cl1	87.29 (6)	C20—C14—H14	120.0
N1—Rh1—Cl1	90.19 (6)	C16—C15—C20	120.8 (4)
P2—Rh1—Cl1	87.45 (3)	C16—C15—H15	119.6
P1—Rh1—Cl1	171.78 (3)	C20—C15—H15	119.6
Cl2—Rh1—Cl1	90.41 (3)	C15—C16—C17	120.3 (4)
C8—P1—C21	104.72 (15)	C15—C16—H16	119.8
C8—P1—C27	105.37 (14)	C17—C16—H16	119.8
C21—P1—C27	104.93 (15)	C18—C17—C16	120.7 (5)
C8—P1—Rh1	100.60 (9)	C18—C17—H17	119.6
C21—P1—Rh1	111.85 (9)	C16—C17—H17	119.6
C27—P1—Rh1	127.10 (11)	C17—C18—C19	119.2 (4)
C18—P2—C39	108.44 (16)	C17—C18—P2	125.0 (3)
C18—P2—C33	107.38 (14)	C19—C18—P2	115.7 (2)
C39—P2—C33	104.33 (14)	N11—C19—C18	119.6 (3)
C18—P2—Rh1	100.25 (12)	N11—C19—C20	120.5 (4)
C39—P2—Rh1	119.73 (10)	C18—C19—C20	119.8 (3)
C33—P2—Rh1	116.00 (9)	C15—C20—C19	119.1 (5)
F3A—P3—F6A	98.7 (10)	C15—C20—C14	123.3 (4)
F3A—P3—F2A	96.5 (6)	C19—C20—C14	117.6 (4)
F6A—P3—F2A	92.8 (7)	C22—C21—C26	119.1 (4)
F6B—P3—F5B	99.3 (12)	C22—C21—P1	122.0 (3)
F3A—P3—F1A	89.1 (5)	C26—C21—P1	118.8 (3)
F6A—P3—F1A	170.5 (9)	C21—C22—C23	119.6 (5)
F2A—P3—F1A	91.7 (7)	C21—C22—H22	120.2
F6B—P3—F4B	85.3 (8)	C23—C22—H22	120.2
F5B—P3—F4B	87.0 (10)	C22—C23—C24	119.4 (6)
F6B—P3—F3B	109.7 (17)	C22—C23—H23	120.3
F5B—P3—F3B	89.9 (12)	C24—C23—H23	120.3
F4B—P3—F3B	165.0 (17)	C25—C24—C23	120.0 (5)
F3A—P3—F4A	163.5 (12)	C25—C24—H24	120.0
F6A—P3—F4A	93.0 (9)	C23—C24—H24	120.0
F2A—P3—F4A	94.5 (11)	C24—C25—C26	120.5 (6)
F1A—P3—F4A	78.3 (11)	C24—C25—H25	119.8
F6B—P3—F2B	73.3 (16)	C26—C25—H25	119.8
F5B—P3—F2B	169.0 (18)	C25—C26—C21	121.4 (5)
F4B—P3—F2B	100.3 (14)	C25—C26—H26	119.3
F3B—P3—F2B	85.1 (12)	C21—C26—H26	119.3
F6B—P3—F1B	158.1 (19)	C32—C27—C28	119.0 (3)
F5B—P3—F1B	98.6 (18)	C32—C27—P1	120.4 (3)
F4B—P3—F1B	83.2 (15)	C28—C27—P1	120.6 (3)
F3B—P3—F1B	82.8 (16)	C29—C28—C27	120.7 (4)
F2B—P3—F1B	90.6 (15)	C29—C28—H28	119.7
F3A—P3—F5A	76.1 (9)	C27—C28—H28	119.7
F6A—P3—F5A	84.0 (6)	C30—C29—C28	119.7 (4)
F2A—P3—F5A	171.3 (9)	C30—C29—H29	120.1
F1A—P3—F5A	92.7 (7)	C28—C29—H29	120.1
F4A—P3—F5A	93.7 (10)	C31—C30—C29	120.3 (4)
C2—N1—C9	118.3 (3)	C31—C30—H30	119.8
C2—N1—Rh1	122.6 (2)	C29—C30—H30	119.8
C9—N1—Rh1	118.45 (18)	C30—C31—C32	120.7 (5)
C12—N11—C19	119.0 (3)	C30—C31—H31	119.7
C12—N11—Rh1	121.9 (2)	C32—C31—H31	119.7
C19—N11—Rh1	119.2 (2)	C27—C32—C31	119.6 (4)
Cl4A—C1A—Cl3A	104.2 (8)	C27—C32—H32	120.2
Cl4A—C1A—H1A	110.9	C31—C32—H32	120.2
Cl3A—C1A—H1A	110.9	C34—C33—C38	119.2 (3)
Cl4A—C1A—H1B	110.9	C34—C33—P2	120.3 (2)
Cl3A—C1A—H1B	110.9	C38—C33—P2	120.5 (2)
H1A—C1A—H1B	108.9	C33—C34—C35	120.2 (3)
C1B—O1B—H1F	109.5	C33—C34—H34	119.9
O1B—C1B—H1C	109.5	C35—C34—H34	119.9
O1B—C1B—H1E	109.5	C36—C35—C34	120.1 (3)
H1C—C1B—H1E	109.5	C36—C35—H35	120.0
O1B—C1B—H1D	109.5	C34—C35—H35	120.0
H1C—C1B—H1D	109.5	C37—C36—C35	119.9 (3)
H1E—C1B—H1D	109.5	C37—C36—H36	120.0
N1—C2—C3	123.5 (3)	C35—C36—H36	120.0
N1—C2—H2	118.2	C36—C37—C38	121.2 (3)
C3—C2—H2	118.2	C36—C37—H37	119.4
C4—C3—C2	119.5 (3)	C38—C37—H37	119.4
C4—C3—H3	120.2	C37—C38—C33	119.4 (3)
C2—C3—H3	120.2	C37—C38—H38	120.3
C3—C4—C10	119.5 (3)	C33—C38—H38	120.3
C3—C4—H4	120.2	C40—C39—C44	118.6 (3)
C10—C4—H4	120.2	C40—C39—P2	122.4 (3)
C6—C5—C10	121.5 (3)	C44—C39—P2	119.0 (2)
C6—C5—H5	119.3	C39—C40—C41	119.7 (4)
C10—C5—H5	119.3	C39—C40—H40	120.2
C5—C6—C7	121.1 (3)	C41—C40—H40	120.2
C5—C6—H6	119.5	C42—C41—C40	121.5 (4)
C7—C6—H6	119.5	C42—C41—H41	119.3
C8—C7—C6	120.3 (3)	C40—C41—H41	119.3
C8—C7—H7	119.9	C41—C42—C43	120.1 (4)
C6—C7—H7	119.9	C41—C42—H42	120.0
C7—C8—C9	119.2 (3)	C43—C42—H42	120.0
C7—C8—P1	124.0 (2)	C42—C43—C44	119.4 (4)
C9—C8—P1	116.7 (2)	C42—C43—H43	120.3
N1—C9—C8	118.9 (3)	C44—C43—H43	120.3
N1—C9—C10	120.8 (3)	C43—C44—C39	120.8 (4)
C8—C9—C10	120.3 (3)	C43—C44—H44	119.6
C4—C10—C5	124.1 (3)	C39—C44—H44	119.6
			
C9—N1—C2—C3	0.1 (5)	C13—C14—C20—C15	178.9 (3)
Rh1—N1—C2—C3	−170.8 (3)	C13—C14—C20—C19	−0.5 (5)
N1—C2—C3—C4	−1.2 (6)	C8—P1—C21—C22	11.2 (3)
C2—C3—C4—C10	1.7 (6)	C27—P1—C21—C22	121.9 (3)
C10—C5—C6—C7	−1.6 (8)	Rh1—P1—C21—C22	−96.9 (3)
C5—C6—C7—C8	−0.1 (7)	C8—P1—C21—C26	−172.8 (2)
C6—C7—C8—C9	2.6 (6)	C27—P1—C21—C26	−62.0 (3)
C6—C7—C8—P1	−173.1 (3)	Rh1—P1—C21—C26	79.2 (2)
C21—P1—C8—C7	77.0 (3)	C26—C21—C22—C23	0.1 (5)
C27—P1—C8—C7	−33.4 (3)	P1—C21—C22—C23	176.2 (3)
Rh1—P1—C8—C7	−166.8 (3)	C21—C22—C23—C24	0.1 (6)
C21—P1—C8—C9	−98.7 (2)	C22—C23—C24—C25	−0.7 (8)
C27—P1—C8—C9	150.9 (2)	C23—C24—C25—C26	1.1 (8)
Rh1—P1—C8—C9	17.4 (3)	C24—C25—C26—C21	−0.8 (7)
C2—N1—C9—C8	−179.0 (3)	C22—C21—C26—C25	0.2 (5)
Rh1—N1—C9—C8	−7.6 (4)	P1—C21—C26—C25	−175.9 (3)
C2—N1—C9—C10	0.4 (5)	C8—P1—C27—C32	−73.9 (3)
Rh1—N1—C9—C10	171.7 (2)	C21—P1—C27—C32	175.8 (3)
C7—C8—C9—N1	176.0 (3)	Rh1—P1—C27—C32	42.6 (4)
P1—C8—C9—N1	−8.1 (4)	C8—P1—C27—C28	103.4 (3)
C7—C8—C9—C10	−3.4 (5)	C21—P1—C27—C28	−6.9 (3)
P1—C8—C9—C10	172.6 (3)	Rh1—P1—C27—C28	−140.1 (3)
C3—C4—C10—C5	176.4 (4)	C32—C27—C28—C29	0.2 (6)
C3—C4—C10—C9	−1.2 (6)	P1—C27—C28—C29	−177.2 (4)
C6—C5—C10—C4	−176.9 (4)	C27—C28—C29—C30	−1.2 (7)
C6—C5—C10—C9	0.7 (7)	C28—C29—C30—C31	1.1 (9)
N1—C9—C10—C4	0.2 (5)	C29—C30—C31—C32	0.0 (9)
C8—C9—C10—C4	179.5 (3)	C28—C27—C32—C31	0.9 (7)
N1—C9—C10—C5	−177.6 (3)	P1—C27—C32—C31	178.3 (4)
C8—C9—C10—C5	1.7 (5)	C30—C31—C32—C27	−1.0 (8)
C19—N11—C12—C13	1.5 (4)	C18—P2—C33—C34	171.2 (3)
Rh1—N11—C12—C13	−176.6 (2)	C39—P2—C33—C34	−73.8 (3)
N11—C12—C13—C14	0.2 (5)	Rh1—P2—C33—C34	60.1 (3)
C12—C13—C14—C20	−0.7 (5)	C18—P2—C33—C38	−7.1 (3)
C20—C15—C16—C17	−0.6 (7)	C39—P2—C33—C38	107.8 (3)
C15—C16—C17—C18	1.1 (7)	Rh1—P2—C33—C38	−118.2 (2)
C16—C17—C18—C19	−0.4 (5)	C38—C33—C34—C35	−1.2 (5)
C16—C17—C18—P2	−178.5 (3)	P2—C33—C34—C35	−179.6 (2)
C39—P2—C18—C17	−47.8 (3)	C33—C34—C35—C36	0.0 (5)
C33—P2—C18—C17	64.4 (3)	C34—C35—C36—C37	0.8 (5)
Rh1—P2—C18—C17	−174.1 (3)	C35—C36—C37—C38	−0.4 (5)
C39—P2—C18—C19	134.0 (2)	C36—C37—C38—C33	−0.8 (5)
C33—P2—C18—C19	−113.8 (2)	C34—C33—C38—C37	1.6 (4)
Rh1—P2—C18—C19	7.8 (2)	P2—C33—C38—C37	180.0 (2)
C12—N11—C19—C18	178.8 (3)	C18—P2—C39—C40	−13.0 (3)
Rh1—N11—C19—C18	−3.0 (3)	C33—P2—C39—C40	−127.2 (3)
C12—N11—C19—C20	−2.7 (4)	Rh1—P2—C39—C40	100.9 (3)
Rh1—N11—C19—C20	175.5 (2)	C18—P2—C39—C44	164.7 (3)
C17—C18—C19—N11	177.8 (3)	C33—P2—C39—C44	50.5 (3)
P2—C18—C19—N11	−4.0 (4)	Rh1—P2—C39—C44	−81.3 (3)
C17—C18—C19—C20	−0.8 (5)	C44—C39—C40—C41	0.8 (6)
P2—C18—C19—C20	177.5 (2)	P2—C39—C40—C41	178.6 (3)
C16—C15—C20—C19	−0.6 (6)	C39—C40—C41—C42	0.1 (7)
C16—C15—C20—C14	−180.0 (4)	C40—C41—C42—C43	−1.2 (7)
N11—C19—C20—C15	−177.2 (3)	C41—C42—C43—C44	1.3 (7)
C18—C19—C20—C15	1.3 (4)	C42—C43—C44—C39	−0.3 (6)
N11—C19—C20—C14	2.2 (4)	C40—C39—C44—C43	−0.7 (6)
C18—C19—C20—C14	−179.3 (3)	P2—C39—C44—C43	−178.5 (3)
